# Repeated practice runs during on-snow training do not generate any measurable neuromuscular alterations in elite alpine skiers

**DOI:** 10.3389/fspor.2022.829195

**Published:** 2022-07-29

**Authors:** Marine Alhammoud, Olivier Girard, Clint Hansen, Sébastien Racinais, Frédéric Meyer, Christophe André Hautier, Baptiste Morel

**Affiliations:** ^1^Aspetar–Orthopaedic and Sports Medicine Hospital, Doha, Qatar; ^2^French Ski Federation, Annecy, France; ^3^School of Human Sciences (Exercise and Sport Science), The University of Western Australia, Crawley, WA, Australia; ^4^Department of Neurology, Christian-Albrechts-Universität zu Kiel Medizinische Fakultat, Kiel, Germany; ^5^Digital Signal Processing Group, Department of Informatics, University of Oslo, Oslo, Norway; ^6^Inter-University Laboratory of Human Movement Biology, University Claude Bernard Lyon 1, Lyon, France; ^7^Inter-University Laboratory of Human Movement Biology (EA 7424), Savoie Mont Blanc University, Chambéry, France

**Keywords:** winter sport, surface electromyogram, injury prevention, muscle fatigue, alpine ski, elite athlete, variability

## Abstract

**Background:**

Alpine skiers typically train using repeated practice runs requiring high bursts of muscle activity but there is little field-based evidence characterizing neuromuscular function across successive runs.

**Purpose:**

To examine the impact of repeated ski runs on electromyographic activity (EMG) of the knee extensors and flexors in elite alpine skiers.

**Methods:**

Nineteen national team alpine skiers were tested during regular ski training [Slalom (SL), Giant Slalom (GS), Super Giant Slalom and Downhill (Speed)] for a total of 39 training sessions. The surface EMG of the *vastus lateralis* (VL), *rectus femoris* (RF), *vastus medialis* (VM), *biceps femoris* (BF) and *semimembranosus/semitendinosus* (SMST) muscles was continuously recorded along with right knee and hip angles. The EMG *root mean square* signal was normalized to a maximal voluntary contraction (%MVC). The first and fourth runs of the training session were compared.

**Results:**

There was no meaningful main effect of run on EMG relative activation time or mean power frequency beyond the skier's intrinsic variability. However, EMG activity of the *vastii* increased from the first to the fourth run in SL [VM, ~+3%MVC for IL and outside leg (OL), *p* = 0.035)], speed (VL, IL:+6%/OL:+11%, *p* = 0.015), and GS (VM, IL:0/OL:+7%, *p* < 0.001); the later with an interaction with leg (*p* < 0.001) due to a localized increase on the OL. The run time and turn time did not change from the first to the fourth run. There were no meaningful changes in angular velocities, amplitude of movement, or maximal and minimal angles.

**Conclusion:**

Neuromuscular activity remains highly stable in elite skiers with low variability across four runs.

## Introduction

Alpine ski racers face multiple constraints during racing, such as event-specific gate setups and variable terrain conditions that require well-developed skiing skills and physical abilities (White and Johnson, 1993). Course performance (run time) in alpine skiing is multifactorial including tactical (Federolf, 2012; Cross et al., 2021a), biomechanical (Meyer, 2012; Hébert-Losier et al., 2014), kinetic/kinematic (Supej and Holmberg, 2019), and neuromuscular requirements (Berg et al., 1995; Hintermeister et al., 1995; Berg and Eiken, 1999; Turnbull et al., 2009). Alpine skiing requires high eccentric and quasi-static loads on the knee extensors to sustain very large radial forces during the turns (Berg and Eiken, 1999; Kröll et al., 2015b; Alhammoud et al., 2020). Therefore, it is established that elite skiers must generate high leg force levels (Berg et al., 1995; Hintermeister et al., 1995; Kröll et al., 2015a,b; Steidl-Müller et al., 2018) to control their speed appropriately when they are close to their “velocity barrier,” above which they make trajectory mistakes (Supej et al., 2011). Alpine skiing is described as a symmetrical sport due to repeated bidirectional turns (Turnbull et al., 2009). However, the load distribution of lateral forces is about 80% on the outside ski from 50 to 70% of the turn in GS. This is due to a progressive increase of the forces on the outside leg (OL) while the load on the inside leg (IL) remains constant (Meyer et al., 2019). Force measurements have been used to gain insight into the skier's technique such as load evolution during turns and force distribution between leg sides (Meyer et al., 2019). A higher radial force production was explained by a greater capability of the lower limbs to produce total force whereby a greater proportion of this force was applied radially (i.e., in an efficient manner). The skiers with superior physical and technical abilities also select strategies that enable them to minimize dissipation of energy regardless of the trajectory (Meyer and Borrani, 2018; Cross et al., 2021a). An efficient skiing technique requires a well-developed technical ability to apply force onto the snow. It is therefore worthwhile to study the underpinning neuromuscular aspects of the force output in skiers, for each body side independently. Surface electromyogram (EMG) is a useful non-invasive tool to describe the on-snow neuromuscular strategy employed by skiers, which has received little attention so far at elite level (Berg et al., 1995; Hintermeister et al., 1995; Berg and Eiken, 1999; Raschner et al., 2001; Kröll et al., 2005a).

A competitive alpine ski run typically lasts ~45 s to ~2 min (White and Wells, 2015; Stöggl et al., 2018). Importantly, ski runs are repeated several times at near-maximal intensity (Zeglinksi et al., 1998) with the primary goal of on-field training to improve the skier's technique. The literature on the neuromuscular adaptations occurring with the repetition of run in elite skiers is limited and showed either neuromuscular alterations post-skiing or an increase in completion time and rate of incomplete runs while skiing in SL and GS (Tomazin et al., 2008; White and Wells, 2015). Force application remains reproducible on-snow in elite skiers following similar trajectory at the same velocity through imposed gates with very stable performance (Meyer et al., 2019; Cross et al., 2021b). However, available surface EMG literature have mainly focused on fatigue manifestation in recreational to experienced skiers (Casale et al., 2003; Kröll et al., 2005b, 2011; Ushiyama et al., 2005; Akutsu et al., 2008; Kiryu et al., 2011), questioning relevance of these findings to elite competitors.

Consequently, the aim of this pilot study was to characterize on-field neuromuscular adjustments and EMG variability in elite skiers repeating four high-intensity runs. Our real-world approach involved comparing knee extensors/flexors muscle activity patterns with bilateral leg comparison across three disciplines. We hypothesized that repeating high-intensity runs would induce minimal modifications in the EMG intensity of the thigh muscles, yet without alterations of the athlete's skiing technique and performance.

## Materials and Methods

### Participants

Nineteen French national team alpine skiers participated in this study [8 females (23 ± 2 years, 169 ± 5 cm, and 65.0 ± 4.3 kg) and 11 males (24 ± 5 years, 178 ± 7 cm, and 76.2 ± 7.0 kg)]. The International Ski Federation (FIS) points (the lower the better) were on average 11 ± 6 (Europa Cup *n* = 7; World Cup *n* = 12). The participants followed their regular ski training program, performing in one or several disciplines, with a maximum of two athletes tested per day (Table 1). A total of 39 “training sessions x skier” (triplet with the day of test, skier, and discipline) were recorded, including 12 skier-sessions in SL, 19 in GS, and 8 in Speed regrouping Super Giant Slalom (SG) and Downhill (DH). The participants were provided with medical clearance to compete. A power analysis with a power of 0.80, a type 1 error at 0.05 and an expected effect size of *f* = 0.25 (moderate) indicated a required sample size of 22 skier-sessions (G^*^Power v.3.1.9.7). The study was approved by the local university committee of Lyon, with all participants providing written informed consent. All procedures conformed to the standards of the Declaration of Helsinki.

**Table 1:** Description of the skier-sessions (*n* = 39).

**Triplet**			**Gates**	**Alt.**	**Drop**	**Incline**	**Snow**			**Weather**	**Visibility**	**Air temp.**
**Skier, Date, Discipline**			**(** ***n*** **)**	**start**	**(m)**	**(%)**	**Condition**	**Hardness**	**Surface**			**(**°**C)**
S1	13092016	DH	13	872	240	24.7	fresh	hard	smooth	foggy/cloudy	reduced	8.0 (0.0)
S2	22092016	DH	13	872	240	24.7	changing	hard/soft	smooth	foggy/cloudy	reduced	3.7 (0.6)
S3	22092016	DH	13	872	240	24.7	changing	hard/soft	smooth	foggy/cloudy	reduced	3.7 (0.6)
S4	11092016	SG	18	872	240	24.7	fresh	hard	smooth	clear/sunny	good	5.3 (1.2)
S5	11092016	SG	18	872	240	24.7	fresh	hard	smooth	clear/sunny	good	5.3 (1.2)
S2	12092016	SG	18	872	240	24.7	fresh	hard	smooth	clear/sunny	good	3.7 (2.5)
S6	21092016	SG	20	889	240	22.4	changing	hard/soft	bumpy	foggy/cloudy	reduced	10.3 (0.6)
S3	12092016	SG	18	872	240	24.7	fresh	hard	smooth	clear/sunny	good	3.7 (2.5)
S7	01092017	SL	32	532	78	19.9	fresh	hard	smooth	foggy/cloudy	reduced	4.0 (1.0)
S7	21082017	SL	32	308	89	23.4	changing	soft	bumpy	clear/sunny	good	6.3 (1.2)
S8	21082017	SL	32	308	89	23.4	changing	soft	bumpy	clear/sunny	good	6.3 (1.2)
S8	25082017	SL A	46	428	140	29.2	fresh	hard	smooth	snowy	reduced	1.3 (0.5)
S8	25082017	SL B	17	288	66	35	fresh	hard	smooth	snowy	reduced	1.3 (0.5)
S8	31082017	SL	47	669	199	DM	injected	icy	smooth	cloudy	reduced	5.7 (0.6)
S9	09092016	SL A	28	439*	259*	38.9*	artificial	hard/icy	smooth	clear/sunny	good	2.7 (1.2)
S9	09092016	SL B	30				artificial	hard/icy	smooth	clear/sunny	good	2.7 (1.2)
S10	18092016	SL	44	622	132	29.7	changing	hard	smooth	clear/sunny	good	7.3 (1.5)
S11	25092016	SL	45	620	145	30.3	artificial	hard	bumpy	foggy/cloudy	reduced	7.3 (2.3)
S12	17092016	SL	35	464	161	27	changing	hard	bumpy	foggy/cloudy	good	4.3 (1.5)
S13	23092016	SL	45	604	128	29.7	changing	hard/soft	bumpy	foggy/cloudy	good	10.0 (1.0)
S14	01092017	GS	34	461	247	25.4	fresh	hard	smooth	clear/sunny	good	4.0 (1.0)
S7	01092017	GS	34	461	247	25.4	fresh	hard	smooth	clear/sunny	good	4.0 (1.0)
S7	15082017	GS A	27	523	145	21.2	fresh	hard	bumpy	foggy/cloudy	reduced	1.7 (0.6)
S7	15082017	GS B	15	334	110	39.4	fresh	hard	bumpy	foggy/cloudy	reduced	1.7 (0.6)
S8	24082017	GS	35	482	261	24.9	fresh	hard	smooth	clear/sunny	good	−0.3 (0.6)
S8	31082017	GS	34	467	235	20.9	fresh	hard	smooth	clear/sunny	good	5.7 (0.6)
S15	16082017	GS	22	523	133	11.2	fresh	hard	smooth	foggy/snowy	reduced	4.3 (0.6)
S15	24082017	GS	35	482	261	24.9	fresh	hard	smooth	overcast/sunny	good	−0.3 (0.6)
S15	25082017	GS	25	424	199	29.7	fresh	hard	smooth	snowy	reduced	1.3 (0.5)
S16	17092016	GS	28	465	232	27.3	changing	hard	bumpy	foggy/cloudy	good	4.3 (1.5)
S17	14092016	GS	40	517	292	25.1	changing	soft	bumpy	clear/sunny	good	9.3 (1.5)
S18	15092016	GS	29	480	283	29.5	fresh	hard	smooth	snowy	reduced	1.5 (0.6)
S12	20092016	GS	36	872	240	25.1	changing	hard/soft	bumpy	foggy/cloudy	reduced	8.0 (1.0)
S13	21092016	GS	30	711	223	28.4	changing	hard/soft	bumpy	foggy/cloudy	reduced	10.3 (0.6)
S6	16092016	GS	32	465	268	27.8	changing	hard	smooth	clear/sunny	good	5.3 (0.6)
S17	15092016	GS	29	480	283	29.5	fresh	hard	smooth	snowy	reduced	1.5 (0.6)
S11	20092016	GS	36	872	240	25.1	changing	hard/soft	bumpy	foggy/cloudy	reduced	8.0 (1.0)
S18	10092016	GS	31	450	270	31.4	changing	hard	bumpy	foggy/cloudy	reduced	4.3 (0.6)
S6	14092016	GS	40	517	292	25.1	changing	soft	bumpy	clear/sunny	good	9.3 (1.5)

SL, Slalom; GS, Giant Slalom; SG, Super Giant Slalom; DH, Downhill; Alt, altitude; Air t°, air temperature; Drop : vertical drop (m); Incline : mean slope incline in percent. DM: missing data.^*^mean value for the global course (A + B). Triplet: athletes performed competition-style runs in different disciplines on separate days (i.e., on different slopes and course settings); some skiers were tested once, other multiple times. Thus, a given skier performing a GS on a specific day was considered as a different triplet than the same skier performing a GS (or another discipline) on another day, representing a total of 39 “training sessions x skiers” (triplet with the day of testing, skier, and discipline).

### General procedure

The data collection occurred during regular in-field practice (years: 2016–2017; location: Cerro Castor, Ushuaïa, Argentina). Each training session included an average of 6.2 ± 1.3 runs for SL, 5.5 ± 0.5 for GS, and 5.5 ± 0.8 for Speed. For standardization purposes, due to uneven number of runs between the days of testing, runs 1 and 4 were always compared (or runs 2 and 5 in case of an incomplete first or fourth run when the skier did not finish the run). This choice was arbitrary as the competitions consisted of two runs in technical disciplines and one run only in speed disciplines. It was also in the lower range of a training session that generally includes at least 4 runs, and sometimes up to 12 runs in technical disciplines, or 8 runs in speed disciplines. The surface EMG activity of the right leg was synchronously monitored as well as hip and knee angles. The runs were timed and course settings matched the FIS regulation. The visibility was either good (51%, sunny/clear weather) or reduced (49%, snow falling or overcast); the track was smooth (62%) or bumpy (38%); the snow was qualitatively described by the coaches as fresh (49%), changing (41%), artificial (8%) or injected (3%) but mainly hard with regular sleepers between skiers (Table 1). The average run duration was 34.9 ± 8.5 s in SL, 47.8 ± 9.6 s in GS, and 49.0 ± 8.4 s in Speed. All subjects followed a routine indoor warm-up upon arrival in the ski resort followed by ~45 min to 1 h of free skiing, as reported elsewhere (Alhammoud et al., 2021a) with the rotation time between runs of ~20 min including ~9 min of chairlift.

### Measurements

#### Electromyography

Wireless surface EMG electrodes (Trigno, Delsys, Boston, USA) were positioned on the *vastus medialis* (VM), *rectus femoris* (RF), *vastus lateralis* (VL), *biceps femoris* (BF), and *semimembranosus/semitendinosus* (SMST) muscles of the right leg according to the SENIAM recommendations (Hermens et al., 2000). The skin was shaved and cleaned with an alcohol swab before electrodes placement. The data were recorded at 1,926 Hz with a gain of 300 by a portable datalogger (TPM, Delsys, Boston, USA). The raw EMG signal was filtered with a second-order Butterworth bandpass filter (20–500 Hz). Artifacts were automatically removed if the EMG intensity exceeded an upper and lower EMG intensity threshold computed as the *root mean square* (RMS) mean ± 6 × SD. Following this, the cycles containing signals with large artifacts—typically caused by transient and random vibrations inherent to alpine skiing (Supej and Ogrin, 2019)—were visually excluded after inspection. Baseline fluctuation was removed *via* a 1 s moving average. The RMS was calculated over a 125-ms epoch and normalized to an isometric maximal voluntary contraction (MVC). For the normalization of the quadriceps (VL, RF, and VM), the participants performed an isometric knee extension in a sitting position with the knee at 110° and the hip at 90° (180° representing full extension). For the hamstrings (BF and SMST), the participants performed a knee flexion in prone position with the knee at 135° (180° representing full extension). The force was recorded *via* a strain gauge (S-type, Interface, Scottsdale, Arizona, USA) and the maximum RMS activity was defined as the highest 500 ms of the EMG signal during the force plateau. At least three knee extensions and three knee flexions were performed and only trials within 10% of the maximal force recorded during the procedure were averaged as the reference value representing the maximal neural drive obtained during MVC tests.

#### Performance and goniometry

The performance and kinematic parameters were reported to give a perspective to the potential neuromuscular changes. Indeed, the disciplines of neurophysiology and physics (mechanics) provide a neuromechanical perspective on the study of human movement (Enoka, 2008). The primary macroscopic performance parameter was the run time, measured using an approved FIS wireless system composed of a starting-gate wand and a dual-beam photocell at the final gate of the course. Knee and hip angles of the right leg were monitored through electrogoniometers (SG 150, Biometrics Ltd, Newport, UK) at 148 Hz following the procedures detailed elsewhere (Alhammoud et al., 2020, 2021b). The goniometer had a resolution of +0.1° in a range of 180°, a repeatability of 1° and an accuracy ±2° measured over a range of 90°. Following visual inspection of the amplitude spectral density and Bode plots (Alhammoud et al., 2020), the angles were low-pass filtered (fourth-order Butterworth, cut-off frequencies of 0.5, 1, 2, and 2.5 Hz for DH, SG, GS, and SL, respectively) to remove the higher frequency domain data such as noise and vibrations (Nemec et al., 2001; Spörri et al., 2017). The power spectral density peaks of acceleration at the shank of elite skiers below 4 Hz are most likely related to the frequency of GS/SL turns and/or to the skier's movements (Spörri et al., 2017). The higher frequencies would have likely been dampened by the musculotendinous structures (Mester, 1997) and by the joints (Fasel et al., 2016; Spörri et al., 2017). Indeed, the knee joint attenuates the signal power of nearly all occurring vibrations in both GS and SL for elite skiers, while the hip joint dampens the vibrations particularly at frequencies <10 Hz (Spörri et al., 2017). The angle data were derived to compute the joint angular velocity.

### Calculations

#### Cycle and leg determination

**Figure 1:**
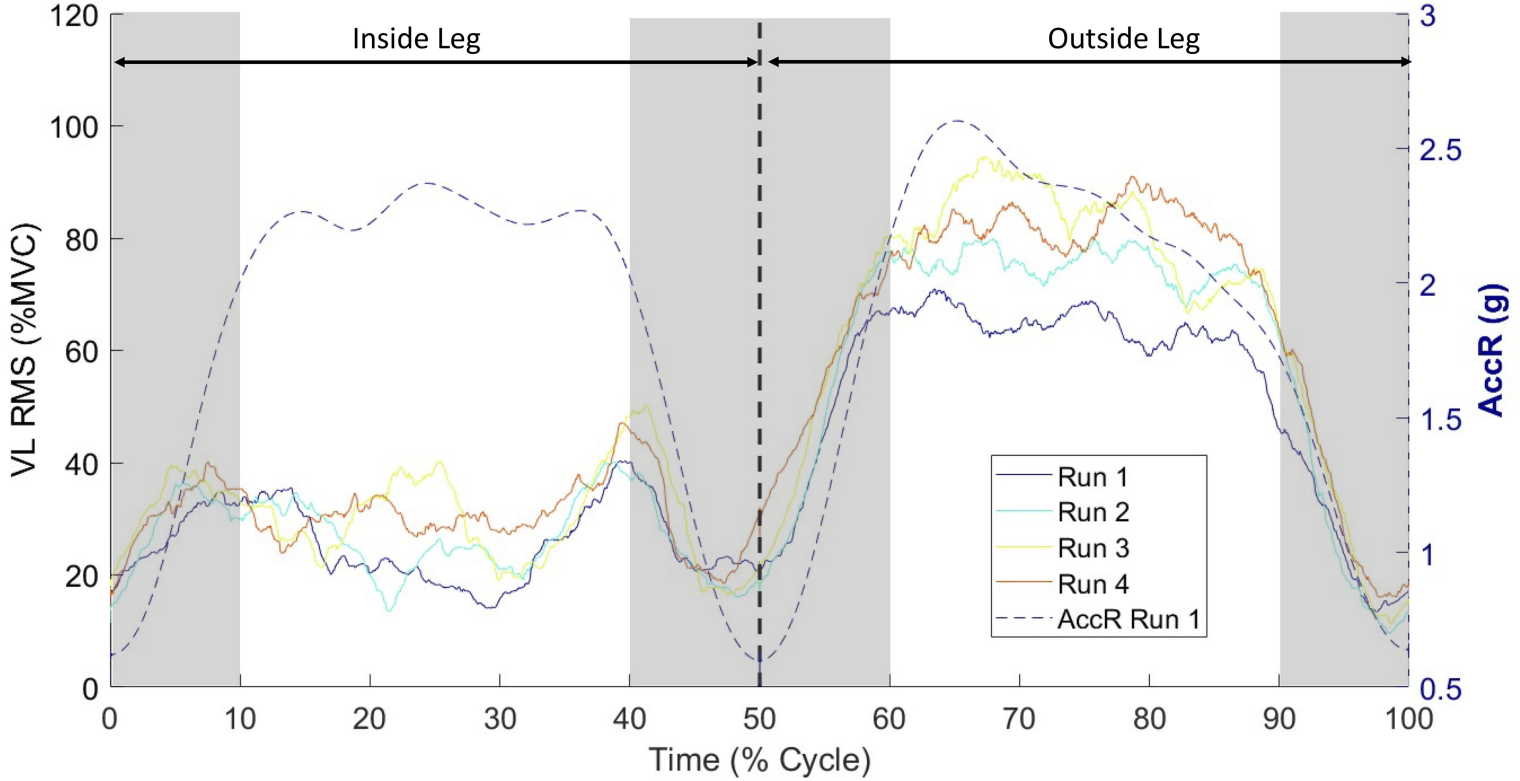
Illustration of a time-normalized ski cycle. This double turn is composed of a right turn (i.e., inside leg, IL, 0–50%) and a left turn (i.e., outside leg, OL, 50–100%), for the EMG activity in *vastus lateralis* (VL). Example from one World Cup skier during a session of Giant Slalom from run one to four. Each run represents the average of all cycles within a run (minus the first and last cycles). The shaded gray zones representing the edge changing phases (“unloading” phases ranged 0–10, 40–60, and 90–100% of the cycle) were discarded from the EMG analyses. MVC, maximum voluntary contraction; RMS, EMG amplitude in *root mean square* value over 125-ms epoch. The turn switch is materialized by the black dotted line at 50% of the cycle, at the minima of the resultant acceleration (AccR).

The TPM data logger was located on the skier's stomach, under the racing suit and included a 3-axial accelerometer recording at 148 Hz, from which the resultant acceleration (AccR) was determined: $AccR\;\left(g\right)=\sqrt{x^{2}+y^{2}+z^{2}}$. The AccR was low-pass filtered with a fourth-order Butterworth, cut-off frequencies of 0.8, 1, 2, and 3 Hz for DH, SG, GS, and SL, respectively. The minimal inflection point of the AccR signal derivative was used to identify the turn switch (Nakazato et al., 2011; Kröll et al., 2015b; Spörri et al., 2018) and then the signals were automatically time-normalized to 100% of each leg (Alhammoud et al., 2020). A cycle represented a right turn (IL) plus a left turn (OL), separated by the edge changing phases (between the ranges 0–10, 40–60, and 90–100% of the cycle; Figure 1) (Nakazato et al., 2011). The knee/hip extension and flexion phases were determined from the knee/hip goniometer.

#### EMG variables

The relative EMG amplitude (RMS %MVC) was computed on the IL and OL for the five muscles after removal of the edge changing phases. The mean power frequency (MPF) was obtained with Fast Fourier Transform. A low-pass filtered EMG (fourth-order Butterworth, cut-off frequencies of 1, 2, 4, and 7 Hz for DH, SG, GS, and SL, respectively) was used to create a linear envelop for the burst duration computation. The EMG activation time was defined as the period where the EMG linear envelope was above 20% of the maximum RMS activity recorded during the MVC (Dorel et al., 2012). The activation time was expressed in absolute time (ms) and relative proportion of the turn (%).

#### Performance and kinematic variables

The time of each run (chrono) was used as an indicator of performance stability. The turn time (ms) was computed for each leg. The minimal and maximal angles for the right knee and hip were used to compute the amplitude of movement and determine the peak joint angular velocity during the flexion and extension phases.

### Statistical analysis

Signal postprocessing was performed through a specifically developed Matlab routine (Matlab 2017a) and statistical comparisons were coded in R (R Foundation for Statistical Computing, Vienna, Austria). A generalized linear mixed model for non-Gaussian response variables distributions was fitted using *glmmTMB* package (Brooks et al., 2017). The EMG activity, angle-related parameters, and turn time were compared between the runs using a generalized linear mixed model with a random effect on the skier-session (ID) and two fixed effects (run with two levels—first and last—and leg with two levels, IL/OL) to evaluate the inter-run effect. For the amplitude and run time parameters, only the run fixed effect was used as the entire cycle (IL + OL) was analyzed. A random intercept model on the skier-session was used within each discipline and for each signal [dependent variable ~ run + leg + run^*^leg + (1|ID)]. Based on Akaike Information Criteria and log likelihood (Nakagawa et al., 2017), an algorithm automatically determined the best adjustment for the variables after comparison of normal, lognormal, or gamma distributions. Each model was adjusted based on the best distribution for the considered dependent variable.

Significance was set at *p* < 0.05. The marginal and conditional R^2^ were reported as dimensionless standardized effects statistics for linear and generalized linear mixed-effects models (Appendices 1, 2) (Nakagawa et al., 2017; Zhang, 2017). Marginal R^2^ (R^2^m) is concerned with variance explained by fixed factors while conditional R^2^ (R^2^c) is concerned with variance explained by both fixed and random factors. The differences between corresponding R^2^m and R^2^c values reflect how much variability is in random effect (skier-session) compared to the fixed effects (Nakagawa and Schielzeth, 2013) but no cut-off values are available to interpret the magnitude of the effect sizes (ES). As effect sizes for generalized linear mixed models, conditional and adjusted intraclass correlation coefficient (ICC) were computed (Nakagawa et al., 2017), where the grouping (random) factor was the skier-session that has been measured repeatedly as follows:

- ICC_adj_ = random effect variance/(random effect variance + residual variance)
- ICC_cond_ = random effect variance/(random effect variance + residual variance + fixed effect variance)

The random effect variance represents the variability between skier-sessions. The residual variance represents the variability of the measure for a given skier-session. The fixed effect variance is the variability of the fixed effect (run, leg, or both effects). As R^2^m + ICC_cond_ = R^2^c, ICC is another way to present effect sizes obtained with R^2^, that varies between 0 and 1. The more ICC is nullified, the less the variability between the skier-sessions is important compared to the variability of the measures for a given skier-session (and compared to the variability of fixed effects for ICC_cond_). When ICC is close to 1, the dependent variables are more homogenous for a given skier-session than between skier-sessions, and the predictors have no significant effects if we consider ICC_cond_ (see Table 2 for examples of interpretation). To interpret the significant tests, ICC_adj_ and ICC_cond_ were compared: 1) if ICC_adj_ ≈ ICC_cond_, the fixed effect was not large enough (the adjunction of the fixed effect variance to the denominator does not changes the value) and despite a significant *p*-value, it cannot be concluded that there is an effect of the factor on the variable of interest (or the effect is not large enough to be considered; 2) if ICC_adj_ < < ICC_cond_ (large difference), we use the cut-off values of Koo and Li (2016) adapted in our context: ICC_cond_ < 0.5 very strong fixed ES, 0.5 < ICC_cond_ < 0.75 strong fixed ES, 0.75 < ICC_cond_ < 0.9 moderate fixed ES, ICC_cond_ > 0.9 small fixed ES.

**Table 2:** *Post hoc* tests of EMG variables and examples of interpretation of ICC as effect sizes for generalized linear mixed models.

**Variable**	**Discipline**	**Signal**	**Factor**		***P*** **value**		**Effect size**			
			**Leg**	**Run**	**Run**	**Leg**	**R^2^m**	**R^2^c**	**ICC_**adj**_**	**ICC_**cond**_**
**RMS**	SL	VL	IL	—	0.959	NA	0.000	0.999	0.999	0.999
**(%MVC)**			OL	—	0.254	NA	0.013	0.999	0.999	0.986
*example a*			—	First	NA	**0**	0.239	0.999	0.999	0.761
			—	Last	NA	**0**	0.177	1.000	1.000	0.823
	GS	VM	IL	—	0.976	NA		0.999	0.999	0.999
*example b*			OL	—	**0**	NA	0.043	1.000	1.000	0.957
			—	First	NA	**0**	0.521	1.000	1.000	0.479
			—	Last	NA	**0**	0.524	1.000	1.000	0.476
		SMST	IL	—	0.463	NA	0.002	0.992	0.992	0.990
			OL	—	**0.033**	NA	0.023	0.998	0.998	0.975
			—	First	NA	**0**	0.854	0.999	0.993	0.145
			—	Last	NA	**0**	0.895	0.999	0.990	0.104
**Burst**	GS	SMST	IL	—	**0**	NA		1.000	1.000	1.000
**duration**			OL	—	**0**	NA	0.036	1.000	1.000	0.964
**(ms)**			—	First	NA	**0**	0.681	1.000	1.000	0.319
*example c*			—	Last	NA	**0**	0.782	1.000	1.000	0.218
	Speed	VM	IL	—	**0.007**	NA	0.069	1.000	1.000	0.931
			OL	—	0.661	NA	0.009	1.000	1.000	0.991
			—	First	NA	**0**	0.400	1.000	1.000	0.600
			—	Last	NA	**0**	0.131	1.000	1.000	0.869
	SL	RF	IL	—	0.535	NA	0.001	0.472	0.471	0.471
*example d*			OL	—	**0**	NA	0.021	0.491	0.481	0.471
**Burst**	SL	RF	—	First	NA	**0**	0.206	0.527	0.404	0.321
**duration**			—	Last	NA	**0**	0.135	0.574	0.508	0.439
(%)	GS	SMST	IL	—	0.444	NA	0.001	0.530	0.530	0.530
			OL	—	**0.011**	NA	0.006	0.531	0.528	0.525
			—	First	NA	**0**	0.276	0.564	0.398	0.288
			—	Last	NA	**0**	0.339	0.569	0.348	0.230

*SL, Slalom; GS, Giant Slalom; SG, Super Giant Slalom; DH, Downhill; IL, inside leg; OL, outside leg; NA, not applicable; ICC_adj_, adjusted intraclass correlation coefficient; ICC_cond_, conditional intraclass correlation coefficient; R^2^m, marginal squared; R^2^c, conditional R-squared; significant p-values (p < 0.05) are indicated in bold, trend (0.05 ≤ p ≤ 0.01) in italic and bold*.

***Example a:** For RMS of VL in SL, leg effect: ICC_cond_ = 0.761, ICC_adj_ =0.999, p ≈ 0. The fact that ICC_adj_ approaches 1 shows that RMS is more homogenous for a given skier-session than between skier-sessions. Besides, ICC_cond_ < ICC_adj_ and the addition of leg fixed effect diminishes ICC of roughly 25%. Hence, the effect size of the leg on RMS of VL in SL is moderate, nearly strong*.

***Example b:** For RMS of VM in GS, run effect on OL: ICC_cond_ = 0.957, ICC_adj_ = 0.999, p ≈ 0. The fact that ICC_adj_ approaches 1 shows a greater variability between the skier-sessions compared with measures done on the same skier-session. Besides, ICC_cond_ is slightly lower than ICC_adj_. Despite a significant p-value, we can therefore conclude that the effect size of the run on OL on RMS for VM in GS is very small*.

***Example c:** For the absolute burst duration of SMST in GS, leg effect on the last run: ICC_cond_ = 0.218, ICC_adj_ = 1.000, p ≈ 0. The value of ICC_adj_ indicates that there is no variability for this variable in the measurements of a given skier-session. Additionally, the fact that ICC_cond_ < ICC_adj_ confirms the significance of the test namely there is a very strong leg effect on the fourth run for this variable*.

***Example d:** For the relative burst duration of RF in SL, run effect on OL: ICC_cond_ = 0.471, ICC_adj_ = 0.481, p ≈ 0. The value of ICC_adj_ indicates that there is nearly as much variability in the measurements of this variable between skier-sessions than for a given skier-session. Additionally, ICC_cond_ ≈ ICC_adj_, consequently despite a quasi-null p-value, we cannot conclude that there is a run effect of this variable on OL*.

The values R^2^ and ICC represent effect sizes, unlike the coefficient of variation (CV). Three sources of variance were computed in the mixed linear models to describe the data reliability (Appendices 1, 2): (1) the random variance (CV_random_) represents the variance of the data due to the random factor (skier-session), namely, the dispersion of the measures between the skier-sessions; (2) the intra-athlete variance (CV_intra_) represents the dispersion of the data for a given skier-session (or “within-group variance”); (3) the systematic variance (CV_syst_): the higher the CV_syst_, the higher the chance that run or leg factors would likely be significant (if CV_random_ and CV_intra_ are also high).

The estimated marginal means and confidence intervals at 95% (95%CI) were calculated with *emmeans* package (Lenth, 2019) to take into account the repeated measures and were represented with rainclouds (Allen et al., 2019). The raincloud plots were used as a tool to visualize raw data (a point per turn), probability density (cloud), and key summary statistics (i.e., mean ± 95% CI). Rainclouds allow data visualization with high levels of accuracy and transparency to convey the key aspects of statistical effects and raw data with a minimal distortion (Allen et al., 2019). The data were expressed as mean ± SE in the text.

## Results

### EMG activation time

**Figure 2:**
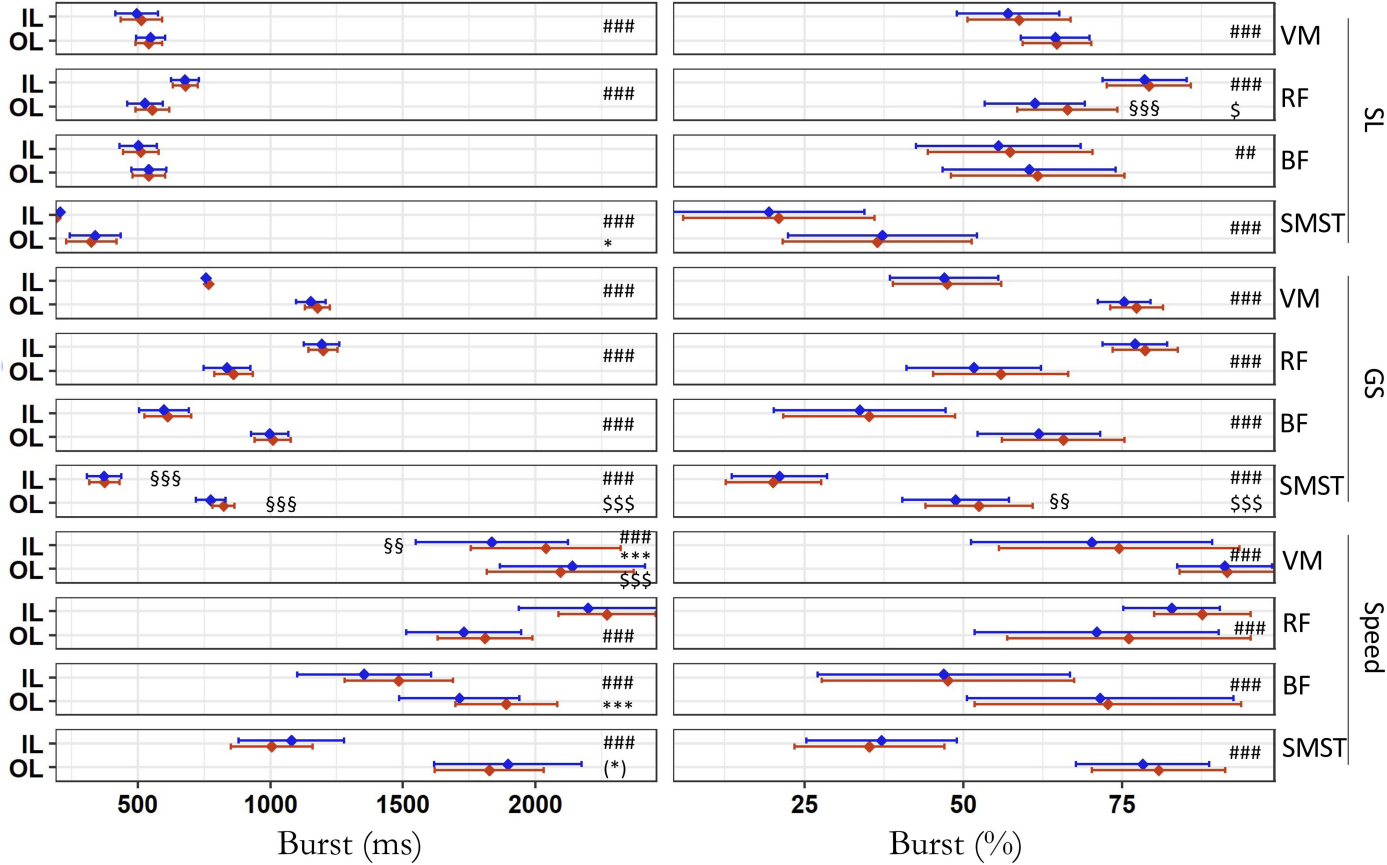
Activation time during the first (blue) and last (red) run across all skier-sessions (*n* = 39). Left: absolute values in ms. Right: relative values in percentage of the turn duration. Values are mean and 95% confidence intervals for the inside (IL) and outside (OL) leg. SL, Slalom; GS, Giant Slalom; Speed, Super Giant Slalom and Downhill; VM, *vastus medialis*; RF, *rectus femoris*; BF, *biceps femoris*; SMST, *semi-membranosus – semi tendinosus*. Due to the similarities between both *vastii*, only the medial head is represented. # difference between IL and OL, * difference between first and last run, $ interaction effect, §*Post hoc* of the interaction showing the difference between first and last run on a single leg. ***, ###, $$$, §§§*p* < 0.001; §§, ## *p* < 0.01; *, $ *p* < 0.05; (*) *p* < 0.10. The raincloud plot visualizes raw data (one point per right and left turn, i.e., inside and outside leg, respectively), probability density (cloud), and key summary statistics of the mean ± 95% confidence intervals. Each skier-session counts a certain amount of turns (right turn, inside leg; left turn, outside leg) composing run one and four. Each individual dot represents the value of the variable for a given right and left turn (inside and outside leg, respectively). The dotted line in blue and red between the two rainclouds of each discipline's subplot links the inside and outside legs of the first and last run, respectively.

The total number of extracted scalar values (i.e., for left and right turns) for EMG signals per muscle was 744 ± 23, 979 ± 13, and 220 ± 0 in SL, GS, and Speed, respectively. The absolute activation time (burst in ms) increased from the first to the fourth run for VM and BF in Speed, for SMST in SL and for VL in GS (all *p* ≤ 0.024, 0.18 < ICC_cond_ < 0.84, moderate to very strong ES; Figure 2; Table 3). There was also an interaction between the run and leg for the SMST in GS, due to a small (ICC_cond_ = 0.96) but significant increase on the OL only (+49 ± 19 ms, *p* < 0.001, R^2^m = 0.036, R^2^c = 1.0) (Figure 2; Table 2). All activation times were also dependent on the leg (*p* < 0.001, 0.106 ≤ R^2^m ≤ 0.808, 0.18 < ICC_cond_ < 0.85, moderate to very strong ES) except for the BF in SL (*p* = 0.266). There were higher values of absolute activation time on IL compared to OL for RF and greater values on OL than IL for the other muscles.

**Table 3:** Comparison of EMG burst duration during the first and last run for five muscles.

**Variable**	**Discipline**	**Signal**	***P*** **value**			**Effect size**		***Emmean diff.*** **Last-First [95%CI]**	
			**Run**	**Leg**	Run*Leg	**ICC**_adj_	**ICC**_cond_	**OL**	**IL**
Burst	SL	VM	0.185	**0.001**	0.160	1.000	0.803	−6.3 [−34.4; 21.8]	17.1 [−10.3; 44.5]
duration		VL	0.880	**0.000**	0.113	1.000	0.728	−23.2 [−53; 6.6]	3.3 [−24.2; 30.7]
(ms)		RF	0.844	**0.000**	0.106	1.000	0.335	27.8 [2.3; 53.2]	1.3 [−28.1; 30.7]
		BF	0.524	0.266	0.691	1.000	0.990	0.1 [−26.5; 26.8]	9.3 [−17.3; 35.8]
		SMST	0.024	**0.000**	0.758	1.000	0.771	−14.9 [−43.1; 13.3]	−16.8 [−28; −5.6]
	GS	VM	0.695	**0.000**	0.450	1.000	0.188	25 [−10.4; 60.4]	10 [−31; 51]
		VL	0.018	**0.000**	*0.057*	1.000	0.182	−2.9 [−37.1; 31.2]	39.7 [−2.7; 82.1]
		RF	0.548	**0.000**	0.808	1.000	0.264	24.7 [−22.8; 72.1]	5.5 [−37.7; 48.8]
		BF	0.940	**0.000**	0.242	1.000	0.327	11.8 [−21.1; 44.7]	14.9 [−16.7; 46.6]
		SMST	0.612	**0.000**	**0.000**	1.000	0.245	49.3 [11.4; 87.1]	0.5 [0.5; 0.5]
	Speed	VM	**0.000**	**0.001**	**0.000**	1.000	0.661	−45.4 [−172.6; 81.8]	203.8 [54.9; 352.7]
		VL	0.249	**0.000**	0.546	1.000	0.675	5.5 [−93.1; 104]	60 [−166.7; 286.6]
		RF	0.179	**0.000**	0.717	1.000	0.442	80 [−60; 220.1]	71.2 [−93.9; 236.3]
		BF	**0.001**	**0.000**	0.562	1.000	0.843	176.7 [28.5; 324.8]	130.6 [−21.7; 283]
		SMST	*0.057*	**0.000**	0.268	1.000	0.171	−70.1 [−216.5; 76.4]	−75.5 [−230.3; 79.3]
Burst	SL	VM	0.161	**0.000**	0.368	0.406	0.387	0.3 [−2; 2.5]	1.7 [−1; 4.4]
duration		VL	0.422	**0.000**	0.715	0.319	0.300	0.4 [−1.8; 2.5]	1 [−1.6; 3.6]
(%)		RF	0.582	**0.000**	0.012	0.446	0.366	5.1 [2.6; 7.7]	0.7 [−1.5; 2.9]
		BF	0.175	0.002	0.789	0.723	0.716	1.3 [−1.1; 3.8]	1.8 [−0.9; 4.6]
		SMST	0.330	**0.000**	0.293	0.670	0.616	−0.8 [−4.2; 2.5]	1.6 [−0.9; 4]
	GS	VM	0.728	**0.000**	0.442	0.390	0.249	1.9 [0.4; 3.5]	0.5 [−2.6; 3.5]
		VL	0.162	**0.000**	0.632	0.443	0.276	1.2 [−0.3; 2.7]	1.6 [−1.5; 4.7]
		RF	0.292	**0.000**	0.221	0.431	0.336	4.3 [1.3; 7.2]	1.6 [−0.7; 3.9]
		BF	0.322	**0.000**	0.297	0.621	0.483	3.9 [1.4; 6.3]	1.5 [−1; 4]
		SMST	0.529	**0.000**	0.037	0.374	0.258	3.7 [0.8; 6.5]	−1 [−3.5; 1.5]
	Speed	VM	0.109	**0.000**	0.300	0.554	0.455	0.4 [−3; 3.8]	4.3 [−1.2; 9.9]
		VL	*0.057*	**0.000**	0.456	0.493	0.374	2.5 [−0.1; 5.1]	5.5 [−0.9; 11.9]
		RF	0.191	**0.001**	0.958	0.380	0.353	5.1 [−1.8; 11.9]	4.8 [−1.1; 10.7]
		BF	0.880	**0.000**	0.900	0.619	0.533	1.2 [−4.7; 7.1]	0.7 [−7.5; 8.8]
		SMST	0.617	**0.000**	0.411	0.236	0.127	2.5 [−4.2; 9.3]	−1.9 [−9.8; 6]

*OL, outside leg; IL, inside leg; SL, Slalom; GS, Giant Slalom; Speed, Super Giant Slalom and Downhill; ICC_adj_, adjusted intraclass correlation coefficient; ICC_cond_, conditional intraclass correlation coefficient; Emmean diff.: estimated marginal mean of the difference between the last and first run. See Appendix 2 for detailed R squared values and coefficients of variation. Significant p-values (p < 0.05) are indicated in bold, trend (0.05 ≤ p ≤ 0.01) in italic and bold*.

When expressed relatively to the cycle duration (burst in %), there was no difference between the first and the fourth run for any muscles and disciplines (*p* ≥ 0.057) but there was an interaction run^*^leg for SMST in GS and for RF in SL (*p* ≤ 0.037; Figure 2). These interactions were due to an increase of the relative duration of the burst on the OL albeit not clinically relevant (SMST in GS: +3.7 ± 1.4%, *p* = 0.011, R^2^m = 0.006, R^2^c = 0.531, ICC_cond_ ≈ ICC_adj_, negligible ES; RF in SL: *p* < 0.001, R^2^m = 0.021, R^2^c = 0.491, ICC_cond_ ≈ ICC_adj_, negligible ES), but not on the IL (SMST in GS: −1.0 ± 1.3%, *p* = 0.444; RF in SL: +0.7 ± 1.1%, *p* = 0.535). All relative activation times strongly depended on the leg (*p* < 0.002, ICC_cond_ < 0.72) with greater values of the relative burst duration on IL than OL for RF but an opposite pattern for the SMST, BF, VM, and VL.

### EMG activation level

**Figure 3:**
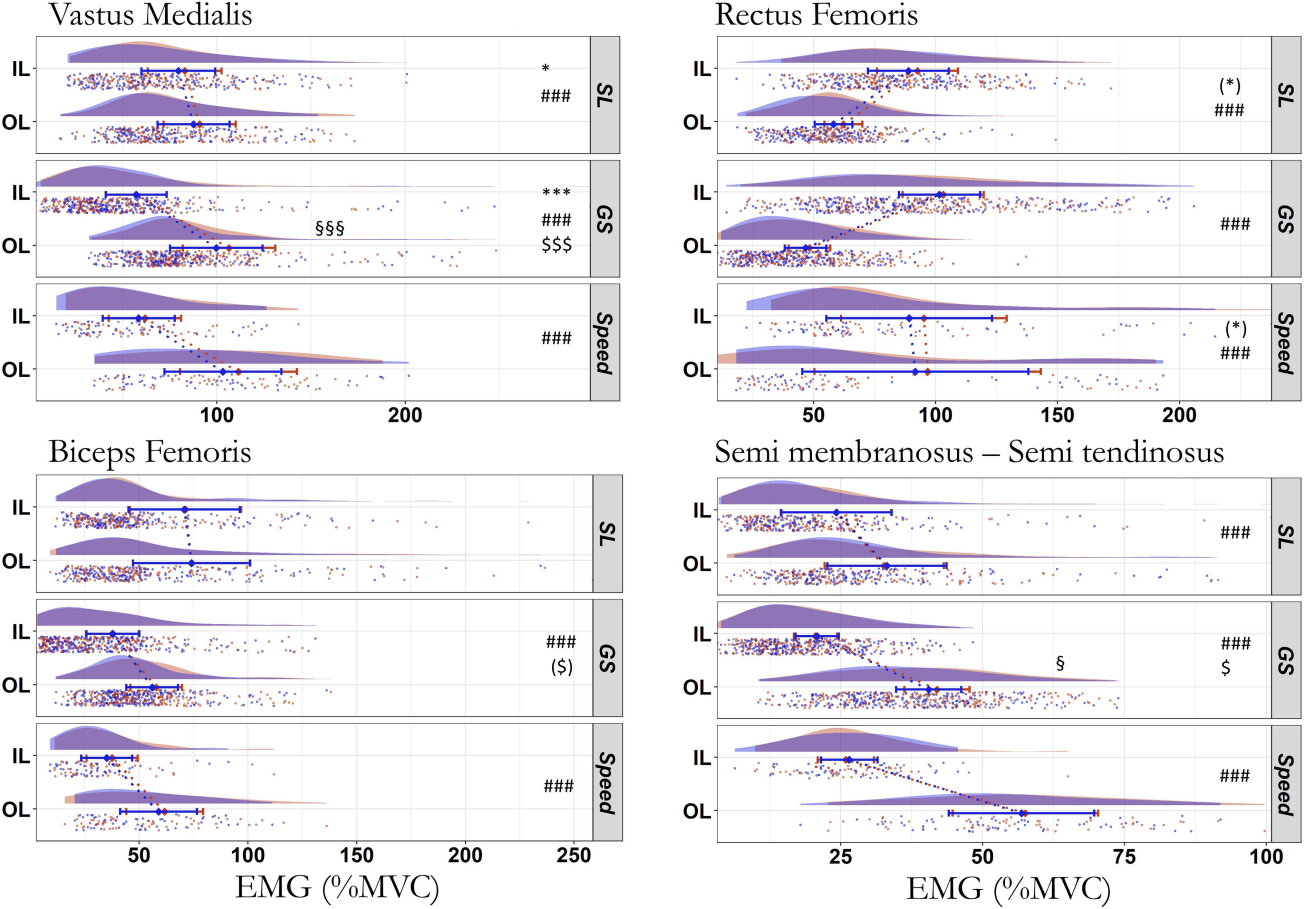
Electromyographic (RMS) activity during the first (blue) and last (red) run across all skier-sessions (*n* = 39). Values are RMS activity in percentage of MVC for the inside (IL) and outside (OL) leg. Due to the similarities between both *vastii*, only the medial head is represented. SL, Slalom; GS, Giant Slalom; Speed, Super Giant Slalom and Downhill. ^#^Difference between IL and OL, * difference between first and last run, $ interaction effect, §*Post hoc* of the interaction showing the difference between first and last run on a single leg. ***, ###, §§§, $$$ *p* < 0.001; *, $, §*p* < 0.05; ($), (*) *p* < 0.10. The raincloud plot visualizes raw data (one point per right and left turn, i.e., inside and outside leg, respectively), probability density (cloud), and the key summary statistics of the mean ± 95% confidence intervals. Each skier-session counts a certain number of turns (right turn, inside leg; left turn, outside leg) composing run one and four. Each individual dot represents the value of the variable for a given right and left turn (inside and outside leg, respectively). The dotted line in blue and red between the two rainclouds of each discipline's subplot links the inside and outside legs of the first and last run, respectively.

The EMG activity increased from the first to the fourth run for the VM in SL (*p* = 0.035, ICC_cond_ = 0.86, moderate ES) and GS (*p* < 0.001, ICC_cond_ = 0.47, very strong ES) and the VL in Speed (*p* < 0.001, ICC_cond_ = 0.22, very strong ES). There was an interaction leg^*^run for the VL in SL and the VM and SMST in GS (*p* ≤ 0.044; Figure 3; Table 4). *Post hoc* analyses (Table 2) revealed that the VM (+6.7 ± 1.2% MVC) and SMST (+1.5 ± 0.7% MVC) increased their activation only in GS and only on the OL (*p* ≤ 0.033, R^2^m ≤ 0.043, R^2^c ≥ 0.998, 0.95 < ICC_cond_ < 0.98, very small ES), while the difference did not reach significance for the VL in SL (OL *p* = 0.254, IL *p* = 0.959). All EMG activities but the BF in SL (*p* = 0.204) depended on the leg with higher values on the OL than IL (*p* ≤ 0.001), except for the RF where the IL activity was higher than the OL activity (*p* < 0.001) in all disciplines. The effects of leg were moderate to very strong (*p* < 0.001, 0.141 ≤ R^2^m ≤ 0.928, 0.07 < ICC_cond_ < 0.86).

**Table 4:** Comparison of EMG activity during the first and last run for five muscles.

**Variable**	**Discipline**	**Signal**	***P*** **value**			**Effect size**		***Emmean diff.*** **Last-First [95%CI]**	
			**Run**	**Leg**	Run*Leg	**ICC**_adj_	**ICC**_cond_	**OL**	**IL**
RMS	SL	VM	0.035	**0.000**	0.943	1.000	0.859	3.2 [0; 6.4]	3.4 [0.2; 6.6]
(%MVC)		VL	0.349	**0.000**	0.044	0.999	0.720	2.3 [−1.7; 6.3]	−0.1 [−4.1; 3.9]
		RF	*0.063*	**0.000**	0.985	0.999	0.239	4.1 [1.3; 6.9]	3.7 [0.7; 6.7]
		BF	0.533	0.204	0.693	1.000	0.997	−0.1 [−1.8; 1.6]	0.6 [−1; 2.2]
		SMST	0.798	**0.000**	0.787	0.997	0.636	−0.5 [−1.9; 0.9]	−0.1 [−0.8; 0.7]
	GS	VM	**0.000**	**0.000**	**0.000**	1.000	0.465	6.7 [4.4; 8.9]	0 [−2.7; 2.8]
		VL	0.367	**0.000**	0.174	0.999	0.286	5.2 [3; 7.4]	0.8 [−1.9; 3.4]
		RF	0.583	**0.000**	0.891	0.998	0.129	1.6 [−0.4; 3.6]	1.5 [−1.9; 4.9]
		BF	0.474	**0.000**	*0.090*	0.999	0.381	1.9 [0.5; 3.2]	−0.3 [−1.1; 0.5]
		SMST	0.441	**0.000**	0.030	0.990	0.100	1.5 [0.1; 2.8]	−0.3 [−1.1; 0.5]
	Speed	VM	0.123	**0.000**	0.125	0.999	0.359	8.3 [2.4; 14.2]	3.3 [−1.7; 8.4]
		VL	0.015	**0.000**	0.278	0.999	0.221	10.6 [4.7; 16.5]	5.6 [0.3; 11]
		RF	*0.078*	**0.000**	0.837	1.000	0.839	5.1 [0.4; 9.8]	6.1 [−1.2; 13.3]
		BF	0.123	**0.000**	0.686	0.998	0.533	2.8 [−0.7; 6.3]	2.5 [−0.8; 5.7]
		SMST	0.591	**0.000**	0.447	0.995	0.071	0.7 [−2.9; 4.2]	−0.6 [−3.6; 2.3]
MPF	SL	VM	0.291	**0.000**	0.407	1.000	0.404	2.3 [0.3; 4.2]	1 [−0.5; 2.5]
(Hz)		VL	0.585	0.007	0.170	1.000	0.871	1.6 [−0.3; 3.5]	−0.6 [−3.3; 2.1]
		RF	0.126	**0.000**	0.309	0.999	0.213	4.6 [2.6; 6.5]	2.2 [0.6; 3.8]
		BF	0.820	0.003	0.841	0.999	0.867	0 [−2.5; 2.5]	0.3 [−1.9; 2.6]
		SMST	0.866	**0.000**	0.959	1.000	0.525	0.4 [−3; 3.7]	−0.2 [−2.7; 2.4]
	GS	VM	*0.082*	**0.000**	0.598	1.000	0.278	2.2 [1; 3.5]	1.4 [−0.3; 3.1]
		VL	0.120	**0.000**	0.967	1.000	0.706	2.7 [1.2; 4.3]	1.6 [−1; 4.2]
		RF	0.003	**0.000**	0.416	0.999	0.542	1.9 [0.2; 3.6]	3 [1.6; 4.3]
		BF	0.871	**0.000**	0.221	0.999	0.147	1.7 [−0.1; 3.6]	−0.2 [−2; 1.6]
		SMST	*0.091*	**0.000**	0.976	1.000	0.333	1.9 [−0.1; 3.9]	1.7 [−0.5; 3.9]
	Speed	VM	0.800	**0.000**	0.337	0.999	0.392	−2.3 [−6.1; 1.5]	0.6 [−2.5; 3.6]
		VL	0.601	**0.000**	0.939	0.999	0.337	0.5 [−2.2; 3.3]	0.9 [−2.6; 4.4]
		RF	0.734	*0.054*	0.563	0.998	0.589	0.8 [−1.6; 3.2]	−0.6 [−2.9; 1.7]
		BF	0.736	**0.000**	0.753	0.996	0.073	−0.2 [−2.8; 2.5]	−0.8 [−3.1; 1.4]
		SMST	0.207	**0.000**	0.328	1.000	0.187	−0.6 [−3.6; 2.5]	2.8 [−1.4; 7.1]

*OL, outside leg; IL, inside leg; SL, Slalom; GS, Giant Slalom; Speed, Super Giant Slalom and Downhill; RMS, root mean square value of the EMG; MPF, mean power frequency; ICC_adj_, adjusted intraclass correlation coefficient; ICC_cond_, conditional intraclass correlation coefficient; Emmean diff.: estimated marginal mean of the difference between the last and first run. See Appendix 2 for detailed R squared values and coefficients of variation. Significant p-values (p < 0.05) are indicated in bold, trend (0.05 ≤ p ≤ 0.01) in italic and bold*.

The CV_intra_ between the first and fourth run among all skiers was 0.6% for RMS (averaged across five muscles and three disciplines). A CV_random_ of 16.8% (range, 9.2–27.8%) for RMS (across five muscles and three disciplines) was found when looking at the repeatability between the skier-sessions (Appendix 2).

### Mean power frequency

**Figure 4:**
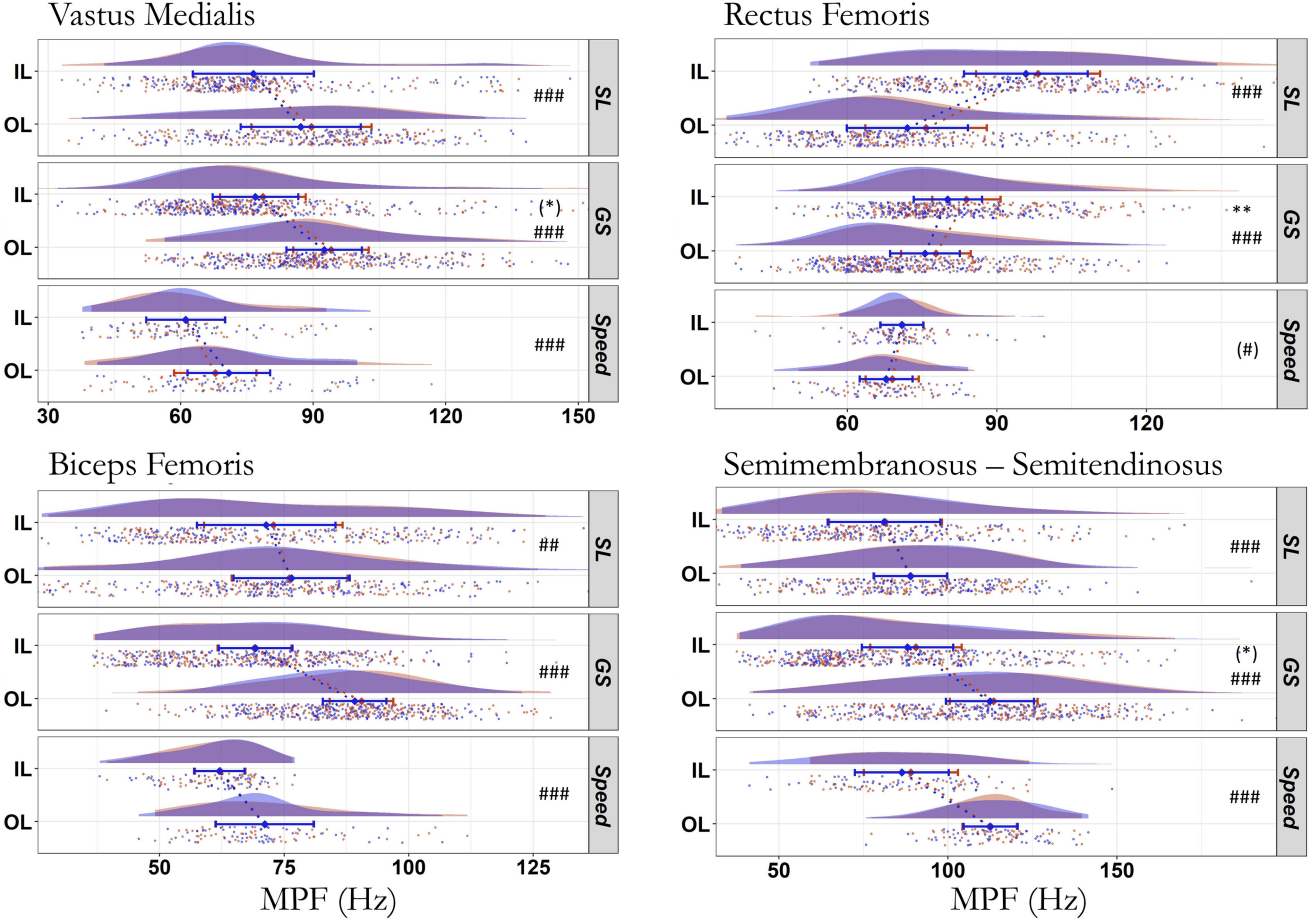
Median Power Frequency (MPF) of the electromyographic activity during first (blue) and last (red) run across all skier-sessions (*n* = 39). MPF values in Hz for the inside (IL) and outside (OL) leg. Due to the similarities between both *vastii*, only the medial head is represented. SL, Slalom; GS, Giant Slalom; Speed, Super Giant Slalom and Downhill. ^#^Difference between IL and OL, *difference between first and last run. ### *p* < 0.001; **, ## *p* < 0.01; (#), (*) *p* < 0.10. The raincloud plot visualizes raw data (one point per right and left turn, i.e., inside or outside leg, respectively), probability density (cloud), and key summary statistics of the mean ± 95% confidence intervals. Each skier-session counts a certain amount of turns (right turn, inside leg; left turn, outside leg) composing run one and four. Each individual dot represents the value of the variable for a given right and left turn (inside and outside leg, respectively). The dotted line in blue and red between the two rainclouds of each discipline's subplot links the inside and outside legs of the first and last run, respectively.

The EMG mean power frequency did not statistically differ between the first and fourth run (*p* ≥ 0.082), except for an increase for the RF in GS (IL: +3 ± 0.7 Hz, OL: +1.9 ± 0.9 Hz; *p* = 0.003, R^2^m = 0.457, R^2^c = 0.999, ICC_cond_ = 0.54, strong ES; Figure 4). There was no significant interaction between run and leg for the five muscles across the three disciplines (*p* ≥ 0.170). There was a main effect of the leg for all disciplines and muscles (*p* ≤ 0.007, 0.129 ≤ R^2^m ≤ 0.927, 0.07 < ICC_cond_ < 0.88, moderate to very strong ES; except for RF in Speed *p* = 0.054), due to the higher values on OL compared to IL for all muscles, but RF where the opposite pattern was seen (IL > OL).

### Performance and kinematic variables

There were no changes in run time (chrono) from the first to the fourth run (*p* > 0.158) except in SL (−0.44 ± 0.14 s; *p* =0.002; Table 5), but this effect was not meaningful (i.e., negligible effect size R^2^m < 0.001, and large variability in the random effect R^2^c > 0.999). There were no changes in turn time from the first to the fourth run (*p* ≥ 0.487; Table 5).

**Table 5:** Run time and turn time.

**Variable**	**Discipline**	***P*** **value**			**Effect size**				**Factor**	**Emmean [95%CI]**		
		**Run**	**Leg**	Run*Leg	**R** ^2^ **m**	**R** ^2^ **c**	**ICC**_adj_	**ICC**_cond_		**First**	**Last**	**Last - First**
Run time	SL	0.002	–	–	**0.001**	0.999	–	–	Run	34.90 [28.04; 41.76]	34.47 [27.61; 41.32]	−0.44 [−0.77; −0.10]
(s)	GS	0.158	–	–	0.010	0.876	–	–	Run	50.96 [47.44; 54.48]	49.56 [46.09; 53.03]	−1.40 [−3.55; 0.75]
	Speed	0.199	–	–	0.007	0.943	–	–	Run	40.87 [39.52; 42.22]	40.59 [39.24; 41.95]	−0.28 [−0.78; 0.23]
Turn time	SL	0.487	0.031	0.478	0.007	0.053	0.050	0.050	OL	787 [754; 821]	805 [771; 841]	−19 [−48; 11]
(ms)									IL	822 [786; 860]	822 [785; 860]	1 [−34; 35]
	GS	0.806	0.385	0.759	**0.001**	0.034	0.030	0.030	OL	1,461 [1,410; 1,515]	1,471 [1,419; 1,525]	−10 [−51; 31]
									IL	1,476 [1,428; 1,526]	1,475 [1,427; 1,525]	1 [−40; 42]
	Speed	0.986	*0.066*	0.586	0.014	0.160	0.150	0.150	OL	2,147 [1,953; 2,360]	2,192 [1,994; 2,410]	−45 [−229; 139]
									IL	2,372 [2,073; 2,713]	2,324 [2,032; 2,658]	48 [−202; 298]

*OL, outside leg; IL, inside leg; SL, Slalom; GS, Giant Slalom; Speed, Super Giant Slalom and Downhill; ICC_adj_, adjusted intraclass correlation coefficient; ICC_cond_, conditional intraclass correlation coefficient; R^2^m, marginal squared; R^2^c, conditional R squared; Emmean: estimated marginal mean. Significant p-values (p < 0.05) are indicated in bold, trend (0.05 ≤ p ≤ 0.01) in italic and bold*.

**Figure 5:**
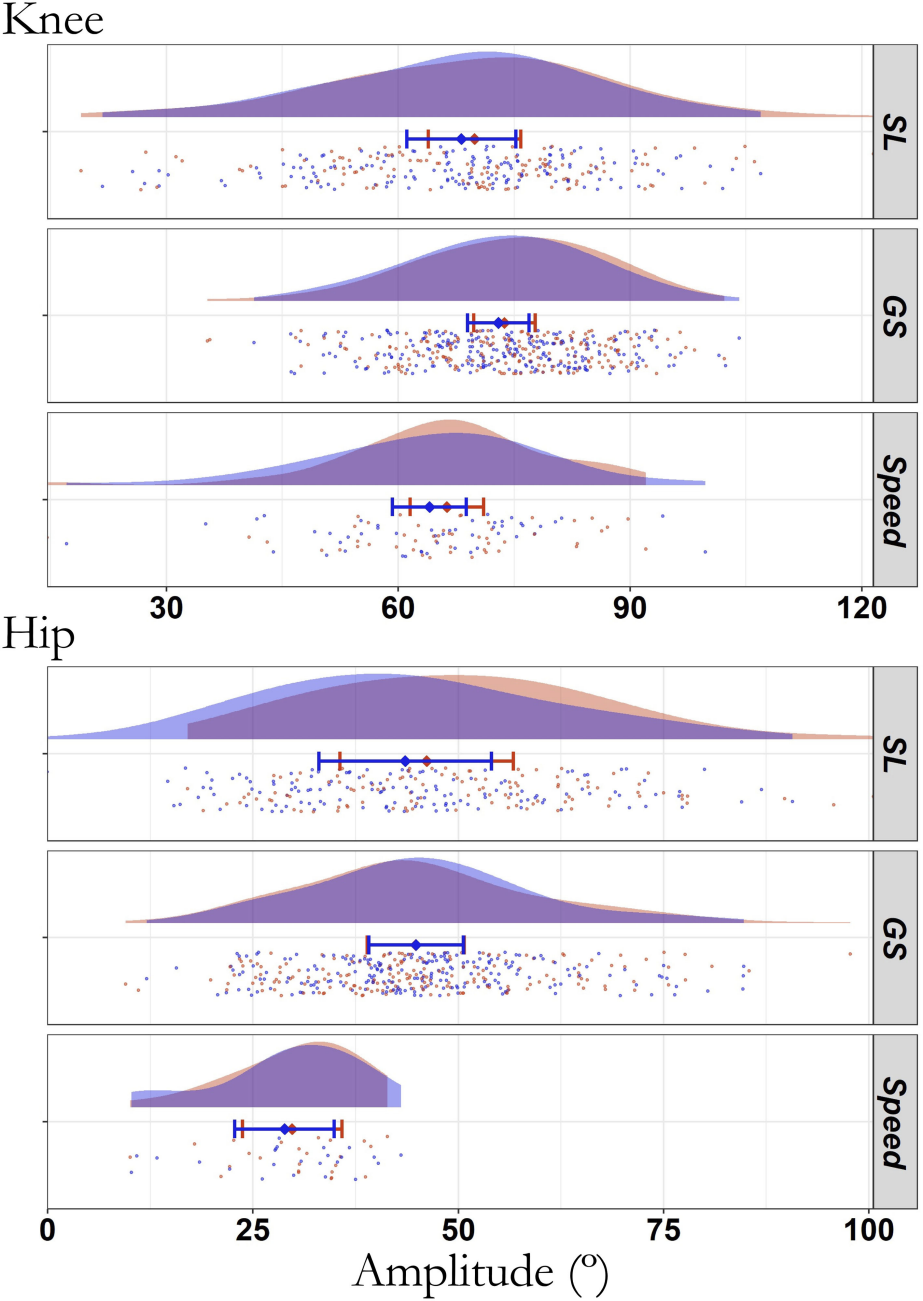
Raincloud of the average amplitude of movement in degrees for the knee and hip (180° full extension) during the first (blue) and last (red) run across all skier-sessions (*n* = 39). SL, Slalom; GS, Giant Slalom; Speed, Super Giant Slalom and Downhill. No statistical differences between first and last run (all *p* ≥ 0.106). The raincloud plot visualizes raw data (a point per cycle), probability density (cloud), and key summary statistics of the mean ± 95% confidence intervals. Each skier-session includes a certain number of cycles (double turns) composing runs one and four. Each individual dot represents the value of the amplitude for a given cycle, i.e., a double turn with an inside plus outside leg turn. SL Slalom, GS Giant Slalom, Speed Super Giant Slalom and Downhill. No statistical differences between first and last (all *p* ≥ 0.106).

**Figure 6:**
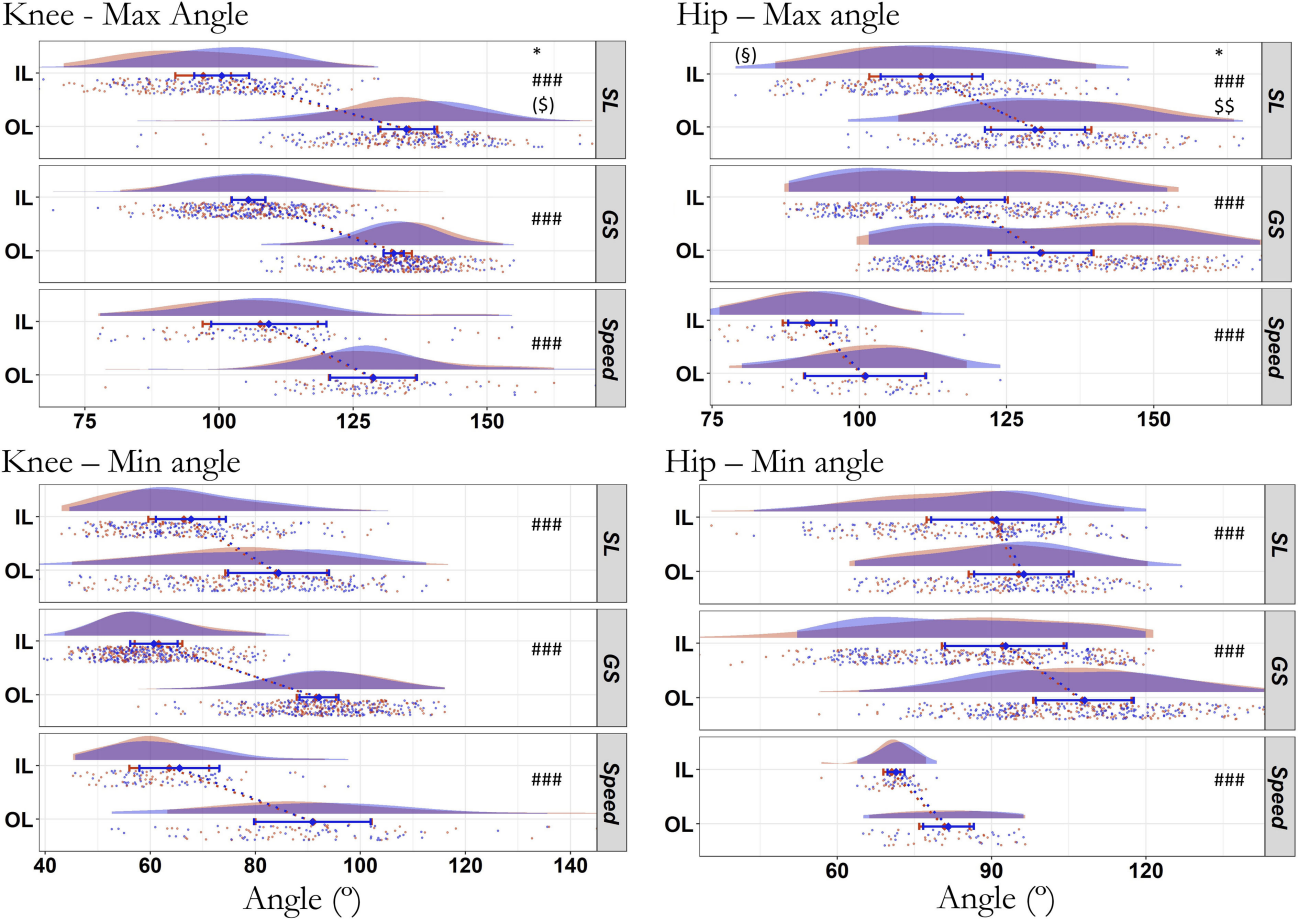
Minimal and maximal angle of the knee and hip during the first (blue) and last (red) run across all skier-sessions (*n* = 39). Values in degrees for the inside (IL) and outside (OL) leg. SL, Slalom; GS, Giant Slalom; Speed, Super Giant Slalom and Downhill. ^#^Difference between IL and OL, *difference between first and last run, $ interaction effect, § *Post hoc* of the interaction showing the difference between first and last run on a single leg. ### *p* < 0.001; $$ *p* < 0.01; * *p* < 0.05; ($), (§) *p* < 0.10. The raincloud plot visualizes raw data (one point per right and left turn, i.e., inside and outside leg, respectively), probability density (cloud), and key summary statistics of the mean ± 95% confidence intervals. Each skier-session counts a certain number of turns (right turn, inside leg; left turn, outside leg) composing run one and four. Each individual dot represents the value of the variable for a given right and left turn (inside and outside leg, respectively). The dotted line in blue and red between the two rainclouds of each discipline's subplot links the inside and outside legs of the first and last run, respectively.

The total number of turns analyzed for each angle-related parameter of the knee/hip (for left and right turns) was 587/485, 859/801, and 216/110 in SL, GS, and Speed, respectively. There were no significant changes in the amplitude of movement from the first to the fourth run for all joints and disciplines (all *p* > 0.106, R^2^m ≤ 0.006; Figure 5). The maximal and minimal angles did not change for any joint in any discipline (*p* > 0.257; Figure 6) except for the maximal knee and hip angles in SL (*p* ≤ 0.024, ICC_cond_ < 0.50, very strong ES). The changes in the maximal hip angle in SL depended on the leg (interaction leg^*^run: *p* = 0.007, R^2^ = 0.354, R^2^c = 0.781), with both non-significant values decrease on the IL (−1.9 ±1.0°, *p* = 0.060) and increase on the OL (+1.1 ± 1.0°, *p* = 0.306). The interaction run^*^leg did not reach significance for the knee (*p* = 0.052; IL: −3.4 ± 1.2° and OL: +0.4 ± 1.2°), despite a decrease in knee maximal angle in IL only (*p* = 0.015, ICC_cond_ = 0.1, very strong ES). There was a strong to very strong fixed ES (ICC_cond_ < 0.68) of leg for all joints angles across all disciplines (*p* < 0.001, 0.136 < R^2^m < 0.973).

**Figure 7:**
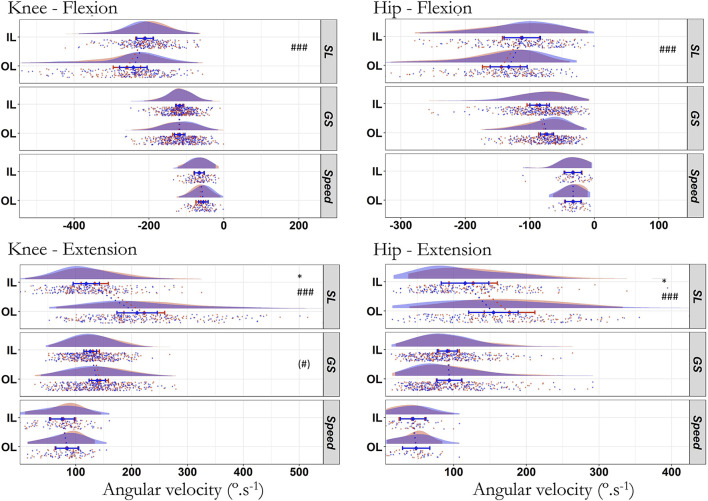
Peak angular velocity of the knee and hip during the first (blue) and last (red) run across all skier-sessions (*n* = 39). Values in degrees per second for the inside (IL) and outside (OL) leg. Due to the similarities between both *vastii*, only the medial head is represented. SL: Slalom, GS: Giant Slalom, Speed: Super Giant Slalom and Downhill. ^#^Difference between IL and OL, *difference between first and last run. ### *p* < 0.001, * *p* < 0.05, (#) *p* < 0.10. A negative angular velocity indicates a knee/hip flexion. The raincloud plot visualizes raw data (one point per right and left turn, i.e., inside or outside leg, respectively), probability density (cloud), and key summary statistics of the mean ± 95% confidence intervals. Each skier-session counts a certain number of turns (right turn, inside leg; left turn, outside leg) composing run one and four. Each individual dot represents the value of the variable for a given right and left turn (inside or outside leg, respectively). The dotted line in blue and red between the two rainclouds of each discipline's subplot links the inside and outside legs of the first and last run, respectively.

The peak angular velocity of the hip and knee did not change from the first to the fourth run during both the extension (*p* > 0.141) and flexion (*p* > 0.482) phases in any joint and discipline (Table 6), except for SL showing a very large increase (ICC_cond_ = 0.17) in knee (IL: 15.2 ± 5.7° s^−1^; OL: 12.8 ±c7.8 s^−1^) and a strong increase (ICC_cond_ = 0.52) in hip (IL: 11.2 ± 5.5 s^−1^; OL: 22.1 ± 7.0 s^−1^) peak extension velocity (*p* ≤ 0.025, R^2^m ≥ 0.482, R^2^c = 1.0). There was also an effect of leg in SL with higher absolute values on OL during the flexion and extension phases (*p* < 0.001; Figure 7) but not in other disciplines.

**Table 6:** Comparison of first and last run for joint kinematics parameters.

**Variable**	**Discipline**	**Signal**	**P value**			**Effect size**		***Emmean diff.*** **Last-First [95%CI]**	
			**Run**	**Leg**	Run*Leg	**ICC**_adj_	**ICC**_cond_	**OL**	**IL**
Min angle	SL	HR	0.283	**0.000**	0.670	1.000	0.583	−1 [−2.9; 0.9]	−0.8 [−3.2; 1.6]
(°)		KR	0.588	**0.000**	0.715	0.999	0.323	−0.4 [−2.8; 1.9]	−1.4 [−3.4; 0.7]
	GS	HR	0.856	**0.000**	0.400	1.000	0.232	−0.5 [−2.3; 1.3]	−0.6 [−1.9; 0.7]
		KR	0.490	**0.000**	0.956	0.998	0.027	−0.5 [−2.3; 1.3]	0.9 [−0.3; 2.1]
	Speed	HR	0.596	**0.000**	0.955	0.994	0.038	−0.8 [−5.2; 3.7]	−0.9 [−2.9; 1.2]
		KR	0.257	**0.000**	0.459	0.999	0.109	0.2 [−5.2; 5.6]	−1.9 [−4.7; 0.8]
Max angle	SL	HR	0.024	**0.000**	0.007	0.661	0.427	1.1 [−1; 3.1]	−1.9 [−3.8; 0.1]
(°)		KR	0.015	**0.000**	*0.052*	0.311	0.100	0.4 [−2; 2.9]	−3.4 [−5.8; −1]
	GS	HR	0.785	**0.000**	0.785	0.789	0.682	0.4 [−1.3; 2]	0.5 [−1.1; 2.1]
		KR	0.941	**0.000**	0.264	0.161	0.049	1.5 [0; 2.9]	−0.1 [−1.7; 1.5]
	Speed	HR	0.684	**0.000**	0.823	0.218	0.169	−0.2 [−4.6; 4.2]	−0.9 [−5.7; 3.9]
		KR	0.438	**0.000**	0.651	0.497	0.341	−0.2 [−4.2; 3.8]	−1.6 [−5.9; 2.7]
AV max	SL	HR	0.482	**0.000**	0.651	1.000	0.675	11.5 [0.7; 22.2]	1.9 [−9.9; 13.6]
flexion		KR	0.653	**0.000**	0.147	1.000	0.631	17.1 [3.4; 30.8]	2.1 [−10.7; 15]
(°.s^−1^)	GS	HR	0.734	0.732	0.522	0.999	0.974	2.5 [−2.7; 7.8]	4.3 [−0.7; 9.3]
		KR	0.596	0.833	0.263	0.999	0.959	4.5 [−3.6; 12.6]	−2.6 [−9.3; 4.1]
	Speed	HR	0.722	0.983	0.645	0.994	0.978	1 [−4.3; 6.3]	−1.1 [−9.7; 7.6]
		KR	0.753	0.273	0.440	0.998	0.747	6.8 [−1.6; 15.1]	1.4 [−7.3; 10.2]
AV max	SL	HR	0.025	**0.000**	0.436	1.000	0.518	22.1 [8.4; 35.8]	11.2 [0.4; 22]
extension		KR	0.008	**0.000**	0.987	1.000	0.174	12.8 [−2.5; 28]	15.2 [4; 26.5]
(°.s^−1^)	GS	HR	0.898	0.916	0.939	0.999	0.999	−0.7 [−6.9; 5.6]	2.2 [−3.5; 7.8]
		KR	0.141	*0.080*	0.890	0.999	0.873	4.8 [−3.1; 12.7]	4.6 [−1.7; 10.8]
	Speed	HR	0.700	0.507	0.829	0.998	0.986	−0.1 [−5.9; 5.7]	−1.6 [−8.9; 5.6]
		KR	0.640	0.190	0.607	0.999	0.861	−1.4 [−10; 7.3]	2.3 [−10; 14.5]
Amplitude	SL	HR	0.106	-	-	0.560	0.560	2.6 [−0.6; 5.8]
(°)		KR	0.379	-	-	0.280	0.280	1.7 [−2.9; 6.3]
	GS	HR	0.963	-	-	0.620	0.620	0 [−2.2; 2.1]
		KR	0.402	-	-	0.390	0.390	0.8 [−1.1; 2.7]
	Speed	HR	0.663	-	-	0.200	0.200	0.9 [−3.4; 5.2]
		KR	0.417	-	-	0.050	0.050	2.2 [−3.2; 7.7]

*AV, angular velocity; KR, right knee; HR, right hip; OL, outside leg; IL, inside leg; SL, Slalom; GS, Giant Slalom; Speed, Super Giant Slalom and Downhill; MPF, mean power frequency; ICC_adj_, adjusted intraclass correlation coefficient; ICC_cond_, conditional intraclass correlation coefficient; Emmean diff.: estimated marginal mean of the difference between the last and first run. See Appendix 1 for detailed R squared values and coefficients of variation. Significant p-values (p < 0.05) are indicated in bold, trend (0.05 ≤ p ≤ 0.01) in italic and bold*.

When calculating the repeatability between two runs (first and fourth), the CV_intra_ was 7.8% and 0.1% among all athletes for maximal and minimal knee angle, respectively (knee and hip averaged across three disciplines). The CV_random_ for the repeatability between the skier-sessions were 7.4 and 4.8% for maximal and minimal knee angle, respectively (knee and hip averaged across three disciplines; Appendix 1).

## Discussion

This study examined the impact of repeated ski runs on leg muscle electromyographic activity in elite alpine skiers for three disciplines. The original hypothesis that minimal EMG activity adjustments would occur with runs repetition to maintain the skier performance and technique was globally supported, as evidenced by the low coefficients of variations. The data showed only a few marginal EMG adjustments (e.g., increase VM activity in SL and GS, increase in VL activity in Speed), without changes in the skier performance (i.e., run time and turn time) from the first to the fourth run. There were no differences in EMG mean power frequency between the first and fourth run (except RF in GS); therefore, the neuromuscular characteristics remain highly stable in elite skiers with low variability across four runs.

### Muscle activity

This study showed some decreases (SMST in SL and GS, VM in Speed) as well as increases (VL in GS, BF in Speed) and in absolute activation time (Figure 2). The latter could suggest a change in the contraction-relaxation cycle with the skiers having more difficulty to relax as runs were repeated. Importantly, the relaxation ability between turns at the right timing is a key performance factor (Pfefferlé, 2014) as muscle de-recruitment permits recovery, enhanced blood flow and efficient movement sequence (Szmedra et al., 2001). Dorel et al. (2012) showed that the longer period of activity induced during sprint in cycling is likely to represent a coordination strategy to enhance the work generated by all the muscle groups. However, the absolute burst duration modifications observed during this study remained inconsistent across muscles, legs and disciplines with opposite variations. Moreover, most of these changes disappeared when the EMG signal was expressed relatively to the cycle duration except marginal increases due to an interaction run^*^leg for RF in SL and for SMST in GS (OL: ~+4–5% relative burst duration; Figure 2; Table 3). Those changes were of low magnitude with negligible ES on the OL for both (*p* ≤ 0.011 but ICC_adj_ ≈ ICC_cond_, Table 2), mainly due to the intra-skier variability (high R^2^c) with low clinical relevance (low R^2^m; Appendix 1). Indeed, in contrast to intermediate skiers, a regular muscle activation pattern (VM and BF) with clear switching between IL and OL has been reported in experienced skiers regardless of number of turns (Kiryu et al., 2011). This is also in line with the observation that the expert skiers were able to maintain their posture despite fatigue occurrence (Akutsu et al., 2008). The ability of athletes to maintain consistency in muscle synergy despite changes in torque and posture was also previously reported in other sports such as highly trained cyclists (Hug et al., 2011). The stability of the neuromuscular pattern in elite skiers is further confirmed in this study by the very low intra-skier variability (Appendix 1), as shown by a CV_intra_ ranging from 0.3 to 1.2% for RMS.

The raincloud representation of the RMS activity (Figure 3) showed that RMS data distribution were skewed (highly concentrated on the left and spread out on the right). This further justified the use of generalized linear mixed models as residuals were not normally distributed but instead close to a gamma law. The statistical analyses showed a main run effect with an increase in RMS for the *vastii* (VM in GS on OL, VL in Speed on both legs, VM in SL on both legs; Table 4), along a trend for RF in SL (*p* = 0.063) and Speed (*p* = 0.078). This emphasizes the key role of the quadriceps in alpine skiing whatever the discipline (Figure 3). We observed a specific increase in medial hamstrings (SMST) on the OL only in GS, but its effect size was small (ICC_cond_ = 0.975; Table 2) with a limited clinical relevance (+1.5% MVC). The limited increases in EMG activity from the first to the fourth run may suggest some neuromuscular adjustments due to peripheral disturbances, although this explanation is less likely as there was enough inter-set recovery time. This is consistent with the fact that central fatigue is considered to recover quickly (Carroll et al., 2017) and was possibly present at the end of each run but subsequently recovered in the 20–30 min period separating the runs.

None of the MPF changes from the first to the fourth run (Figure 4) reached significance in this study (*p* ≥ 0.082 for VM and SMST in GS), except for the RF in GS on both legs where a modest increase was seen (~+2–3 Hz; *p* = 0.003, moderate ES; Table 4). However, the practical implications are not known. Regardless of any “inter-run” effect, it is worth noting that we observed higher values of MPF on the OL compared to IL for all muscles except RF where the opposite pattern was seen (IL > OL), whatever the discipline. This indicates a strong preferential and asymmetrical OL use in alpine ski racing, although an important co-loading of the IL for VL has previously been reported in ski carving (Müller and Schwameder, 2003). The biarticular RF showed a marked EMG intensity and MPF on the IL and also on the OL albeit to a lesser extent. Similarly, previous results show higher myoelectric frequency for the VL and RF on the OL and IL, respectively (Kröll et al., 2011), along with marked differences between legs (Kröll et al., 2010). Interestingly, GS has also been reported to induce a greater muscle oxygen desaturation than SL (Szmedra et al., 2001) as the skiers maintain a lower posture and thus, probably a greater static load with higher percentage of MVC on OL (Figure 3), consistent with compromised blood flow to the VL (Szmedra et al., 2001). Such local hypoxia can impair neural drive during rapid contractions in elite skiers, but this effect could also be blunted by the larger effect of fatigue when contractions are repeated (Alhammoud et al., 2018). The high level of activity of all muscle fibers during alpine skiing could hypothetically induce an ischaemic environment that would result in a decreased neural drive (Ferguson, 2010). Nevertheless, voluntary activation recovers within 3 min of blood flow restoration (Woods et al., 1987), i.e., a duration shorter than the inter-run interval. Thus, while skiing may induce muscle ischemia and hypoxia leading to an intra-run fatigue, no inter-run EMG changes were visible. This absence of changes may partly be related to recovery between runs. It should also be considered that athletes who repeatedly perform high-force intermittent exercise with short rest periods such as skiers or climbers have an adapted vasculature to enhance the blood flow response limiting this fatigue effect (Ferguson and Brown, 1997; Ferguson, 2010). Finally, the absence of inter-run EMG changes may be partly due to a lack of sensitivity of the values such as activation time or average EMG activity. Indeed, the reduction of the turn to one scalar value collapses the temporal aspects of the signal and potentially hinders important information such as shift of EMG activation onset/offset (Figure 1). Since the EMG changes were not large enough to be detected by traditional statistics, more sensitive analyses such as Statistical Parametric Mapping (SPM) (Alhammoud et al., 2019) and wavelet transform should be applied on the entire turns' signal.

In our on-field study, some neuromuscular adjustments (e.g., increases in *vastii* RMS activity) may have resulted from the influence of external factors, such as the evolution of the ski-snow properties. Indeed, the snow conditions changed in 41% of the sessions, generally toward a warmer state (Table 1). While there was minimal track deterioration with regular sleepers between skiers, a softer snow may have induced lower EMG activity levels (Federolf et al., 2009), partly blunting a possible EMG activity increase due to inter-run effect. Indeed, the average vibration intensities decreased by a factor of 2–3 as the snow turned from hard frozen to soft in experienced skiers performing 24 runs, concomitantly with a substantial decrease in the EMG activity of the thigh muscles (Federolf et al., 2009). In any case, those changes were minor when considering the repeatability between two runs (first and fourth) with a CV_intra_ of 0.6% for RMS (averaged across five muscles and three disciplines, Appendices 1, 2) as elite skiers appear to maintain a stable neuromuscular activity across four runs.

### Performance, kinematic, and technical parameters

The elite skiers participating in this study were able to maintain their performance (turn time and run time) across the four runs investigated (Table 5). Remarkably, elite skiers maintained their performance despite eventual changes in track conditions. While the snow surface was generally very hard (Table 1), in reality it is never perfectly rigid. The exact nature of the local snow surface was not precisely assessed and could progressively change with each racer as the snow is scraped and deformed (Reid et al., 2020). A recent kinematic study highlighted the role played by the ski shovel in groove formation during SL carved turns in elite skiers, as the ski continues to penetrate deeper into the snow with each subsequent run (Reid et al., 2020). During smooth carved turns, dynamical effects of the skis such as vibration and body-generated forces by the skier are negligible; but when skiing takes place on a varying terrain with rough and rippled snow surface, vibrational effects likely impact the ski-snow contact pressure (Heinrich et al., 2010). Overall, a combination of sufficient recovery time along proper track and skis preparation would allow elite skiers to maintain their performance for at least four runs during a regular racing training session.

The data from this study showed no measurable adjustments in the amplitude of movement, minimum angle and peak eccentric angular velocity between the first and fourth run in elite skiers (Figures 5–7). Indeed, the raincloud representation of the values showed a close overlap of the first and fourth run data (Figure 5). The absence of modification in flexion velocity suggests that elite skiers were able to maintain their ability to decelerate the downward movement and thus their capacity to resist the gravity and centrifugal force during the carved turn. There was no change in the maximal angle (Figure 6) except in SL for the knee joint only, where a strong main effect of run was seen with a decrease on IL (−3.4°) and negligible increase on OL (<1°). The *post hoc* test of the interaction leg^*^run on the hip did not reach significance. Additionally, the peak extension angular velocity was not affected by runs repetition in Speed and GS. The SL was the only discipline showing some kinematic alterations with runs repetition, albeit the magnitude of change remained small (Figures 6, 7): the decrease (~-3°) of the knee maximal angle on the IL and faster (~+11–22° s^−1^) knee and hip peak extension velocities are hardly visible to the naked eye. In the absence of clear concomitant EMG changes, the small increase of peak extension angular velocity for hip and knee on both legs in SL (Figure 7) could be attributed to external factors such as the ski-snow interaction. These results showed that elite skiers are able to maintain similar kinematic characteristics while repeating runs. Stability of the kinematic pattern in elite skier is further confirmed in this study by a low intra skier-session (CV_intra_ ranging from 0.1 to 9.1%) and between skier-sessions (CV_random_ ranging from 1.4 to 12.8%) variability (Appendix 1). The total variability depended on the disparities between the skier-sessions and showed the good reproducibility of the measurement system as well as a homogenous level and steady technique in elite skiers. Compared to their intermediate counterparts, highly skilled skiers can reproduce the same motion pattern with accuracy (Müller et al., 1998). Of note, previous observations (Kiryu et al., 2011) also showed the adoption of a more upright posture in intermediate but not experienced skiers in a trial (5-min skiing demonstration for 4,000 m) and after repeated alpine ski turns. From a practical point of view, the high stability in performance and kinematic parameters in elite skiers suggests that future scientific studies or equipment testing could be done on a limited number of runs in this specific population.

### Limitations of a field study

This study results should be considered specific to the number of runs, the duration of the work periods, as well as the duration and nature (active *vs*. passive) of the recovery pattern in-between. While the results from this study showed that elite skiers could maintain their performance during four runs, an inter-run effect with less stable neuromuscular pattern could have presumably been observed with additional and/or longer runs, as shorter recovery periods are constrained by the necessity to take the chairlift. It should be acknowledged that the number of runs (*n* = 4) included in this study is in the low range of a typical training session, albeit representative of elite practice. The future reliability studies may provide a better understanding of the contributors to the performance stability in alpine skiing and form a basis of worthwhile improvements in conditioning. As visible on the rainclouds (Figures 3–7), there was also less turns in Speed than GS and SL, due to longer turn duration and fewer gates in the Speed disciplines (SL: 40–60, GS: 25–50, SG: 15–40, and DH: 15–35 turns/run) (Gilgien et al., 2018). Thus, SG and DH (similar training slope, too few runs per specialty) were merged. Nonetheless, even if both specialties are traditionally grouped as the Speed discipline (Neumayr et al., 2003; Cross et al., 2021a)), a separate analysis would be recommended in future studies due to distinct kinematic requirements (Alhammoud et al., 2020).

The session used for normalization procedure of EMG signal took place the day before the ski sessions and the electrodes' location was marked with an indelible marker (Rainoldi et al., 2001), as it was not logistically possible for the athletes to complete it on the same day. The reproducibility of isometric MVC performed in the same conditions between days was tested in a separate experiment and was interpreted as *high* or *very high* (0.749 ≤ ICCs ≤ 0.966). Furthermore, a high level of EMG measures repeatability between days was shown for quadriceps (ICC > 0.70) (Rainoldi et al., 2001) and hamstrings (ICC 0.70–0.89) (Bussey et al., 2018) with this procedure. Additionally, medial hamstrings (SM, ST) were not individualized as it was not possible to attribute the recorded EMG signal to one specific muscle (possible crosstalk effect) without ultrasound to precisely localize these two muscles. The strong passive vibrations, due to uneven surfaces and high speeds involved in alpine skiing, stimulate the muscle spindles and activate a larger number of motoneurons, which in turn elevates the EMG activity and can cause early onset of muscle fatigue (Shinohara, 2005). Filtering was therefore necessary to avoid the contamination of the signal due to vibrations that increase the EMG RMS during a given exercise (Borges et al., 2017). However, a small degree of muscle fatigue could have been masked by the vibration effect on EMG since the ski-snow interaction properties likely changed throughout a training session. Finally, the rotations occurring at the skier's hip joint due to counterrotation and dissociation (LeMaster, 2010) were neglected as the goniometer recorded two planes only. However, both wireless surface EMG electrodes and goniometers were selected to ensure that athletes performed at their peak without any disturbance while skiing to preserve measurement ecological validity (Berg et al., 1995; Kröll et al., 2010; Panizzolo et al., 2013).

## Conclusion

The aim of this study was to characterize the EMG changes in response to repeating four competitive runs in elite skiers. There was an increase in RMS from the first to the fourth run for the *vastii* (on both legs for VL in Speed and VM in SL, VM in GS on OL). There was no meaningful main effect of run on EMG activation time or mean power frequency beyond the skier's variability. Performance, defined as run time and turn time, was not affected and there were no meaningful kinematic changes. As hypothesized, there were no clear adjustments suggesting neuromuscular alterations, and the changes observed could partly be attributed to inter-run variability, or changes in the ski-snow properties. Overall, neuromuscular activity remains highly stable from the first to the fourth run in elite skiers with low variability across runs repetition. This highlights the specificities of alpine skiing with the necessity to take the chairlift (passive recovery) between runs and the limited number of runs performed per session by elite skiers. Importantly, this study investigated the EMG changes within a training session in which the skiers were able to maintain their performance. Future studies should look at the fatigue responses to longer sessions or to repeated sessions on consecutive days.

## Data Availability Statement

The raw data supporting the conclusions of this article will be made available by the corresponding author, upon reasonable request.

## Ethics Statement

The studies involving human participants were reviewed and approved by University of Lyon. The patients/participants provided their written informed consent to participate in this study.

## Author contributions

MA and CAH conceived the study. MA and SR collected the data. MA, CH, and BM analyzed the data. MA, OG, and FM interpreted the data. MA drafted the manuscript. All authors read and approved the final manuscript.

## Conflict of interest

The authors declare that the research was conducted in the absence of any commercial or financial relationships that could be construed as a potential conflict of interest.

## Publisher's note

All claims expressed in this article are solely those of the authors and do not necessarily represent those of their affiliated organizations, or those of the publisher, the editors and the reviewers. Any product that may be evaluated in this article, or claim that may be made by its manufacturer, is not guaranteed or endorsed by the publisher.

## Abbreviations

AccR: resultant acceleration
BF: *biceps femoris*
CV_intra_: intra skier-session coefficient of variation
CV_random_: coefficient of variation due to the random effect between skier-sessions
CV_syst_: systematic coefficient of variation due to the fixed effects
EMG: surface electromyographic signal
ES: effect size
FIS: International Ski Federation
GS: Giant Slalom
ICC_adj_: adjusted intraclass correlation coefficient
ICC_cond_: conditional intraclass correlation coefficient
ID: skier-session
IL: inside leg
MPF: mean power frequency
MVC: maximal voluntary contraction
OL: outside leg
R^2^c: conditional R squared
R^2^m: marginal R squared
RF: *rectus femoris*
RMS: *root mean square* of the electromyographic signal
SE: Standard Error
SL: Slalom
SMST: *semimembranosus/semitendinosus*
Speed: Super Giant Slalom and Downhill disciplines
VL: *vastus lateralis*
VM: *vastus medialis*.
