# Supplementary material for: Repeated practice runs during on-snow training do not generate any measurable neuromuscular alterations in elite alpine skiers
Source: Front Sports Act Living. 2022 Jul 29;4:829195. doi: 10.3389/fspor.2022.829195 (PMC9372580; doi:10.3389/fspor.2022.829195)
Supplement: Supplementary file 1 [file Table_1.DOCX]

**Appendix 1:** EMG comparison of the first and fourth run: variability and inference

| **Variable** | **Discipline** | **Signal** | **R^2^m** | **R^2^c** | **CV_intra_** | **CV_random_** | **CV_syst_** |
| --- | --- | --- | --- | --- | --- | --- | --- |
| **RMS** | SL | VM | 0.141 | 1.000 | 0.34 | 14.62 | 6.22 |
|  |  | VL | 0.280 | 0.999 | 0.37 | 12.10 | 7.74 |
|  |  | RF | 0.760 | 1.000 | 0.36 | 9.16 | 16.61 |
|  |  | BF | 0.003 | 1.000 | 0.44 | 23.71 | 1.44 |
|  |  | SMST | 0.362 | 0.998 | 1.17 | 21.55 | 17.06 |
|  | GS | VM | 0.535 | 1.000 | 0.48 | 22.53 | 25.51 |
|  |  | VL | 0.714 | 1.000 | 0.51 | 16.93 | 27.24 |
|  |  | RF | 0.871 | 1.000 | 0.51 | 12.18 | 32.07 |
|  |  | BF | 0.618 | 0.999 | 0.76 | 20.73 | 26.19 |
|  |  | SMST | 0.899 | 0.999 | 1.12 | 11.13 | 33.81 |
|  | Speed | VM | 0.640 | 1.000 | 0.41 | 17.28 | 23.09 |
|  |  | VL | 0.779 | 1.000 | 0.45 | 14.24 | 27.14 |
|  |  | RF | 0.161 | 1.000 | 0.41 | 27.75 | 12.25 |
|  |  | BF | 0.466 | 0.999 | 0.75 | 18.40 | 17.58 |
|  |  | SMST | 0.928 | 1.000 | 0.69 | 9.30 | 33.27 |
| **MPF** | SL | VM | 0.596 | 1.000 | 0.13 | 6.25 | 7.41 |
|  |  | VL | 0.129 | 1.000 | 0.13 | 6.11 | 2.35 |
|  |  | RF | 0.787 | 1.000 | 0.15 | 5.44 | 10.49 |
|  |  | BF | 0.132 | 0.999 | 0.22 | 7.90 | 3.02 |
|  |  | SMST | 0.475 | 1.000 | 0.16 | 6.84 | 6.38 |
|  | GS | VM | 0.722 | 1.000 | 0.13 | 5.62 | 9.06 |
|  |  | VL | 0.294 | 1.000 | 0.14 | 6.76 | 4.35 |
|  |  | RF | 0.457 | 0.999 | 0.14 | 4.41 | 4.06 |
|  |  | BF | 0.853 | 1.000 | 0.16 | 5.53 | 13.38 |
|  |  | SMST | 0.667 | 1.000 | 0.13 | 7.27 | 10.26 |
|  | Speed | VM | 0.608 | 1.000 | 0.17 | 6.56 | 8.10 |
|  |  | VL | 0.663 | 1.000 | 0.17 | 5.11 | 7.12 |
|  |  | RF | 0.410 | 0.999 | 0.12 | 2.53 | 2.12 |
|  |  | BF | 0.927 | 1.000 | 0.16 | 2.68 | 9.55 |
|  |  | SMST | 0.813 | 1.000 | 0.10 | 5.26 | 10.98 |
| **Burst** | SL | VM | 0.197 | 1.000 | 0.06 | 10.90 | 5.57 |
| **duration** |  | VL | 0.272 | 1.000 | 0.06 | 9.28 | 5.74 |
| **(ms)** |  | RF | 0.665 | 1.000 | 0.05 | 8.87 | 12.68 |
|  |  | BF | 0.010 | 1.000 | 0.07 | 16.32 | 1.74 |
|  |  | SMST | 0.229 | 1.000 | 0.22 | 38.87 | 20.93 |
|  | GS | VM | 0.812 | 1.000 | 0.04 | 11.39 | 23.96 |
|  |  | VL | 0.818 | 1.000 | 0.04 | 12.45 | 26.37 |
|  |  | RF | 0.737 | 1.000 | 0.04 | 12.82 | 21.55 |
|  |  | BF | 0.673 | 1.000 | 0.06 | 22.03 | 30.68 |
|  |  | SMST | 0.755 | 1.000 | 0.09 | 18.88 | 33.92 |
|  | Speed | VM | 0.339 | 1.000 | 0.02 | 14.39 | 10.63 |
|  |  | VL | 0.325 | 1.000 | 0.02 | 15.53 | 11.15 |
|  |  | RF | 0.558 | 1.000 | 0.02 | 15.48 | 17.96 |
|  |  | BF | 0.157 | 1.000 | 0.03 | 22.33 | 9.73 |
|  |  | SMST | 0.829 | 1.000 | 0.03 | 11.32 | 25.67 |
| **Burst** | SL | VM | 0.047 | 0.434 | 20.21 | 16.70 | 5.96 |
| **duration** |  | VL | 0.060 | 0.360 | 19.75 | 13.51 | 6.13 |
| **(%)** |  | RF | 0.179 | 0.545 | 17.03 | 15.28 | 10.77 |
|  |  | BF | 0.009 | 0.725 | 21.97 | 35.46 | 4.17 |
|  |  | SMST | 0.080 | 0.696 | 53.11 | 75.64 | 29.27 |
|  | GS | VM | 0.362 | 0.610 | 24.35 | 19.46 | 23.64 |
|  |  | VL | 0.378 | 0.653 | 24.12 | 21.52 | 25.10 |
|  |  | RF | 0.220 | 0.556 | 25.75 | 22.40 | 18.29 |
|  |  | BF | 0.221 | 0.705 | 35.10 | 44.89 | 30.22 |
|  |  | SMST | 0.310 | 0.568 | 48.93 | 37.81 | 42.29 |
|  | Speed | VM | 0.179 | 0.633 | 17.13 | 19.08 | 12.11 |
|  |  | VL | 0.241 | 0.615 | 18.19 | 17.93 | 14.69 |
|  |  | RF | 0.071 | 0.424 | 24.06 | 18.84 | 8.54 |
|  |  | BF | 0.139 | 0.671 | 31.43 | 40.03 | 21.28 |
|  |  | SMST | 0.461 | 0.588 | 34.28 | 19.04 | 36.12 |

EMG: surface electromyographic signal; RMS: *root mean square* (in % of a maximal voluntary contraction); MPF: mean power frequency; SL: Slalom; GS: Giant Slalom; Speed: Super Giant Slalom and Downhill; VM: *vastus medialis*; VL: *vastus lateralis;* RF: *rectus femoris*; BF: *biceps femoris*; SMST: *semimembranosus/semitendinosus*; R^2^c: conditional R squared; R^2^m: marginal R squared; CV_intra_: intra skier-session coefficient of variation; CV_random_: between skier-sessions coefficient of variation (%); CV_syst_: systematic coefficient of variation (variance due to the fixed effects in %).

For the reliability of EMG data, the systematic variability ranged from CV_syst_=17.2-20.5% except for the MPF which was lower (CV_syst_=7.2%), and the random variability ranged from CV_random_=16.1-27.8% for all variables except for MPF (CV_random_=5.6%). The intra-athlete variability was <0.7% for all variables except for the relative burst duration, which ranged from CV_intra_=25.0-31.7%.

**Appendix 2:** Comparison of the first and fourth run for joint kinematics parameters: variability and inference

| **Variable** | **Discipline** | **Signal** | **R^2^m** | **R^2^c** | **CV_intra_** | **CV_random_** | **CV_syst_** |
| --- | --- | --- | --- | --- | --- | --- | --- |
| **Min angle** | SL | HR | 0.417 | 1.000 | 0.12 | 5.51 | 4.72 |
|  |  | KR | 0.676 | 1.000 | 0.20 | 6.07 | 8.89 |
|  | GS | HR | 0.768 | 1.000 | 0.10 | 6.01 | 10.93 |
|  |  | KR | 0.973 | 1.000 | 0.15 | 3.63 | 21.88 |
|  | Speed | HR | 0.962 | 1.000 | 0.11 | 1.39 | 6.99 |
|  |  | KR | 0.891 | 1.000 | 0.19 | 6.21 | 17.87 |
| **Max angle** | SL | HR | 0.354 | 0.781 | 6.43 | 8.98 | 8.14 |
|  |  | KR | 0.679 | 0.779 | 8.76 | 5.89 | 15.46 |
|  | GS | HR | 0.136 | 0.818 | 6.62 | 12.81 | 5.72 |
|  |  | KR | 0.693 | 0.743 | 7.06 | 3.09 | 11.56 |
|  | Speed | HR | 0.223 | 0.392 | 8.83 | 4.65 | 5.30 |
|  |  | KR | 0.315 | 0.655 | 9.13 | 9.07 | 8.80 |
| **AV max** | SL | HR | 0.325 | 1.000 | 0.30 | 15.36 | 10.24 |
| **flexion** |  | KR | 0.369 | 1.000 | 0.11 | 8.99 | 6.84 |
|  | GS | HR | 0.025 | 0.999 | 0.50 | 14.02 | 2.23 |
|  |  | KR | 0.040 | 0.999 | 0.27 | 8.14 | 1.66 |
|  | Speed | HR | 0.016 | 0.994 | 1.49 | 19.28 | 2.37 |
|  |  | KR | 0.251 | 0.998 | 0.58 | 12.31 | 6.92 |
| **AV max** | SL | HR | 0.482 | 1.000 | 0.28 | 16.00 | 14.60 |
| **extension** |  | KR | 0.826 | 1.000 | 0.21 | 11.49 | 24.36 |
|  | GS | HR | 0.000 | 0.999 | 0.43 | 15.63 | 0.33 |
|  |  | KR | 0.126 | 0.999 | 0.22 | 8.40 | 3.17 |
|  | Speed | HR | 0.012 | 0.998 | 0.91 | 19.03 | 1.99 |
|  |  | KR | 0.138 | 0.999 | 0.45 | 15.04 | 5.77 |
| **Amplitude** | SL | HR | 0.005 | 0.565 | 26.98 | 30.63 | 2.77 |
|  |  | KR | 0.003 | 0.279 | 20.95 | 12.98 | 1.25 |
|  | GS | HR | 0.000 | 0.619 | 19.42 | 24.75 | 0.05 |
|  |  | KR | 0.001 | 0.392 | 12.90 | 10.34 | 0.54 |
|  | Speed | HR | 0.003 | 0.205 | 25.38 | 12.78 | 1.57 |
|  |  | KR | 0.006 | 0.059 | 20.92 | 4.96 | 1.73 |

AV: angular velocity; SL: Slalom; GS: Giant Slalom; Speed: Super Giant Slalom and Downhill; KR: right knee; HR: right hip; R^2^c: conditional R squared, R^2^m: marginal R squared; CV_intra_: intra skier-session coefficient of variation (%), CV_random_: between skier-sessions coefficient of variation (%); CV_syst_: systematic coefficient of variation (variance due to the fixed effects in %).

For the reliability of kinematic data, the average systematic variability was <24.4% (averaged CV_syst_=7.2%), and the random variability was CV_random_<16.1% for all variables (averaged CV_random_=11.1%), The intra-athlete variability was CV_intra_=0.1-9.1% for all variables (averaged CV_intra_=6.0%) except for amplitude, which ranged from CV_intra_=12.9-27.0%.
